# Joint Quality–Reliability Analysis of IRS-Assisted Communications in Presence of Inverse Power Lomax Fading Channel

**DOI:** 10.3390/s26165159

**Published:** 2026-08-14

**Authors:** Aleksey S. Gvozdarev, Roman Yu. Manakhov

**Affiliations:** Department of Intelligent Radiophysical Information Systems (IRIS), P.G. Demidov Yaroslavl State University, Yaroslavl 150003, Russia

**Keywords:** fading, channel, quality, reliability, IRS, Inverse Power Lomax, 89.70.+c, 84.40.Ua, 94A15, 94A40

## Abstract

In this work, we study the joint performance of the quality and reliability in terms of quality–reliability (JQR) performance of an intelligent reflecting surface (IRS)-assisted wireless communication system under severe multipath fading and shadowing. The wireless channel model is given by the Inverse Power Lomax (IPL) fading model, representing a heavy-tailed fading channel, which can describe the hyper-Rayleigh fading and is verified using two different experimentally obtained measurement scenarios, namely, the LTE-case for high-frequency, long-range cellular communications and the device-to-device (D2D) case for lower-frequency short-range communications. For the considered channel model and communication scheme, analytical expressions for the outage probability (a metric related to the reliability) and the average bit error rate for both coherent and non-coherent modulation schemes (metrics associated with the quality of the communication system) are provided. By combining the aforementioned expressions, a unified JQR curve, together with its asymptotic forms in the high signal-to-noise ratio regime and asymptotically large number of IRS elements, is derived. It is proved analytically that the use of IRS with infinite elements can remove fading, while for a finite number of IRS elements, a closed-form signal-to-noise ratio (SNR) penalty factor is presented. The numerical analysis demonstrates that coherent modulations outperform non-coherent ones, higher-order quadrature amplitude modulation (QAM) systems are highly sensitive to the multipath fading, and the LTE-case exhibits better performance compared to the D2D-case for equal settings. Moreover, the joint quality–reliability approach highlights the existence of regions where quality is more preferable than reliability, allowing the allocation of resources based on these regions. All expressions have been verified using Monte Carlo simulations with excellent agreement.

## 1. Introduction

Intelligent reflective surfaces or reconfigurable intelligent surfaces (IRS/RIS) have recently gained widespread use as a paradigm-shifting communication technology, enabling a connection session even in the most severe propagation conditions. Although they are being intensively studied [1] concerning their possible implementation in 6 G [2,3] and beyond 6 G, there is still much work to be done [4,5,6,7,8,9], and several critical issues exist preventing them from being implemented in the up-to-date standards.

In its principal core, IRS is a communication device comprising numerous passive (or quasi-passive) reflecting elements [10,11], which are independently and adaptively tuned by the intelligent controller to perform reflection of the impinging waves with a specific phase-shift [12,13]. The total reflected field (as the result of interference of single-element scattered waves) is defined by the adaptation strategy employed, which is chosen in such a way as to optimize some given quality performance metric (or their combination) at the receiver. Thus, from the most general description of the technology, it is clear that the overall mathematical formalization of the IRS-assisted communication session is heavily dependent upon the channel models between the signal source and the IRS, as well as between the IRS and the receiver [14,15,16]. Furthermore, the adopted mathematical framework defines the overall system performance and, thus, leads to further changes in the link adaptation strategy. The implied recursive logic underpins the critical role of the propagation statistical model. Thus, the closer it is to the real-life channels, the more appropriate the performance results.

Nowadays, propagation channel statistical description is a mature field with a plethora of well-established results [17] ranging from the most simplistic ones [18,19,20,21,22] to the most intricate [23,24,25,26,27,28,29,30,31], which aim to describe as many physical effects impairing signal transmission as possible, including multipath fading, shadowing, multiple reflections, etc. However, as the primary benefit of deploying RIS lies in their application to the cases of poor propagation conditions, the channel models must be chosen accordingly. The severity of such conditions can be quantified in terms of the so-called hyper-Rayleigh regimes (HRRs) (first discussed in [25,32,33,34], strictly defined in [35] asymptotically for infinitely increasing signal-to-noise ratio (SNR), with its further modification for a finite SNR [36,37]). Thus, the chosen model should be capable of handling the hyper-Rayleigh conditions, which correspond to severe fading and shadowing.

Lastly, the choice of the model is governed by its complexity. Although the most intricate models (for instance [24,26,27,30]) cover much of the propagation effects, their complicated mathematical description prevents deriving closed-form (or even exact) results, which are critical for inferring the insights into how the IRS-assisted system performs.

Combining the given requirements imposed on the channel model (i.e., adequacy to measurements, capability of heavy fading description, and analytical tractability), the possible choice sufficiently narrows down. Among the ones that have gained specific attention in recent years, one can mention the Inverse Power Lomax model [22], which is simple enough to admit closed-form representation of most link characteristics and is validated on real-life measurements. Although it is fairly new, it is intensively studied for various applications [38,39] and has shown that it is capable of describing the hyper-Rayleigh behavior of the fading channel. Moreover, it demonstrated good agreement with experimental data obtained for vastly different systems and channels, including both full-scale experiments and synthesized numerical data, higher-frequency long-range cellular communication, and lower-frequency short-range device-to-device communication [22]. This makes IPL a perfect candidate for channel modeling in IRS-assisted communications. Recently, such a combination was adopted for the analysis of the necessary number of IRS elements required to elevate the communication system from the deepest of the hyper-Rayleigh regimes (i.e., full-HRR) to the no-HRR situation. Although some strong analytical results were obtained, the question of the IRS system performance was not raised; thus, the description of the quality and reliability of the IRS-assisted communications with IPL channels remains open. Moreover, as it is widely accepted in communication theory, the standalone metrics (quality or reliability) cannot solely describe the overall system performance. Thus, an alternative approach of joint quality–reliability (JQR) analysis (proposed and elaborated in [38,40]) seems to be a reasonable complement.

To fill the existing gap, the current research assumes a wireless communication system employing the intelligent reflective surface functioning in the presence of multipath fading and shadowing subjected to the inverse power Lomax statistical model from the transmitter to the panel and from the panel to the receiver (i.e., with no line-of-sight propagation). The IPL model parameters were extracted from the experimental data fitting, which correspond to two distinct scenarios: higher-frequency long-range cellular communication (denoted as LTE-case for later) and lower-frequency short-range device-to-device communication (denoted as D2D-case). For both cases, analytical description of the link performance (in terms of the average error rate of coherent and non-coherent modulations) and link reliability (in terms of outage probability) was derived. The obtained results were combined to yield the closed-form joint quality–reliability description.

The major contributions of this work can be summarized as follows:

- Closed-form analytical expressions for the following performance metrics were obtained:
  – Average error rates for coherent and non-coherent modulation schemes of the IRS-assisted communication system with Inverse Power Lomax fading channel models in each of the segments;
  – Reliability metric (i.e., outage probability);
  – Joint quality–reliability curve;
  – Asymptotic expressions for all of the derived metrics (i.e., quality, reliability, and JQR) for infinitely increasing average signal-to-noise ratio and infinitely increasing number of IRS elements.
- Closed-form and numerical demonstration of the fact that the IRS (with an infinitely increasing number of elements) can completely eliminate fading, whereas the finite number of IRS elements can be regarded as a penalty factor for the average signal-to-noise ratio (which is evaluated in closed-form).
- The identified operating regions where the IRS-assisted system prefers quality over reliability and vice versa.
- An extensive comparative numerical analysis of the assumed IRS-assisted communication system metrics for various channel and system parameters demonstrating their relative sensitivities to parameter variations for two experimentally verified distinctive propagation scenarios: higher-frequency long-range cellular communication and lower-frequency short-range device-to-device communication.
- The performance benchmarks and practical recommendations for wireless communications system designers on the choice of system parameters (e.g., number of IRS elements, average SNR, modulation type and format, etc.) that achieve specific target values of quality, reliability, or their joint implication.

The remainder of the research is structured as follows: Section 2 provides the general description of the IRS-assisted communications, the statistical channel model employed, and introduces the adopted system performance metrics; Section 3 presents an overall complete statistical description of the assumed system model; Section 4 presents the derived closed-form and asymptotic results of the system performance; Section 5 contains a thorough numerical analysis of the derived expressions for various system and channel parameter values; Section 6 discusses the limitations of the obtained results and possible directions for future work; and the conclusions are drawn in Section 7.

## 2. Preliminaries

### 2.1. IRS-Assisted Communication System

Let us assume a classical IRS-assisted communication system [12], which connects the single-element transmitter with the single-element receiver via $N_{IRS}$-element intelligent reflective surface situated in the far-field of both the receiver and the transmitter. The propagation between all the nodes exhibits frequency-flat fading with the transmission coefficients denoted as ${\dot{h}}_{1,l}=\left|{\dot{h}}_{1,l}\right|e^{-i\arg\{{\dot{h}}_{1,l}\}}$ and ${\dot{h}}_{2,l}=\left|{\dot{h}}_{2,l}\right|e^{-i\arg\{{\dot{h}}_{2,l}\}}$, where index *l* indicates the *l*th reflecting element $\left(l=1,2,\ldots ,N_{IRS}\right)$ with the reflection coefficient $|{\dot{\rho }}_{l}|e^{i\arg\{{\dot{\rho }}_{l}\}}$. In this case, the instantaneous SNR at the receiver (as indicated in (Equation (11) [10])) can be rewritten as

(1) $$\gamma_{\Sigma }={\left|\sum_{l=1}^{N_{IRS}}|{\dot{h}}_{1,l}\left|\right|{\dot{\rho }}_{l}\left|\right|{\dot{h}}_{2,l}|e^{i(\arg\left\{{\dot{\rho }}_{l}\right\}-\arg\left\{{\dot{h}}_{1,l}\right\}-\arg\left\{{\dot{h}}_{2,l}\right\}}\right|}^{2}{\bar{\gamma }}_{\Sigma }.$$

Let us assume the case of perfectly tuned IRS: lossless (i.e., $|{\dot{\rho }}_{l}|=1$), which completely eliminates the random phase shifts ($\arg\left\{{\dot{\rho }}_{l}\right\}=\arg\left\{{\dot{h}}_{1,l}\right\}+\arg\left\{{\dot{h}}_{2,l}\right\}$), existing due to multipath propagation, by co-phasing them. Thus, from (1), the instantaneous output SNR will be defined in terms of envelope statistics only, that is,

(2) $$\gamma_{\Sigma }={\left|\sum_{l=1}^{N_{IRS}}|{\dot{h}}_{1,l}\left|\right|{\dot{h}}_{2,l}|\right|}^{2}{\bar{\gamma }}_{\Sigma }.$$

For later on, it will be convenient to denote the sum in (2) as *Z*, i.e., $\gamma_{\Sigma }=Z^{2}{\bar{\gamma }}_{\Sigma }$.

It is clear that this assumption limits the effects that impact the system overall performance quality. In real-life applications, one should note that it is virtually impossible to completely eliminate the impact of propagation path phase-shifts due to two main reasons: (1) because of their randomness (for fast fading, the compensation is valid only on average, i.e., in statistical sense), (2) for practical implementations the $\arg\{{\dot{\rho }}_{l}\}$ are commonly chosen to be discrete with a finite (and usually very coarse) step. This causes the existence of the residual phase error, which can be critical for signal constellation rotation, thus, for further symbol detection. Nevertheless, it should be pointed out that there are numerous research projects addressing this issue [41,42,43,44,45]; hence, the solutions are known. As the current research mainly focuses on the upper bound of the performance, which is achieved in the case of perfectly tuned IRS, this assumption will be used for further derivations.

### 2.2. Statistical Channel Model

Let us assume that both subchannels (i.e., between the transmitter and the IRS, as well as between the IRS and the receiver) exhibit fading with the probabilistic model of the inverse power Lomax (IPL) distribution. As was mentioned earlier, such a model can successfully describe long-range, as well as short-range, radio communications [22]. It is a two-parameter distribution (with a scale parameter $\beta \in R_{+}$ and shape parameter $\alpha_{R}\in R_{+}$) with the probability density function (PDF) and cumulative distribution function (CDF) of the signal-to-noise ratio γ defined as follows [22]:

(3) $$f_{\gamma_{i}}\left(\gamma_{i}\right)=\frac{\alpha_{i}\beta_{i}}{{℧}_{\alpha_{i},\beta_{i}}}\frac{{{\bar{\gamma }}_{i}}^{\beta_{i}}}{{\gamma_{i}}^{\beta_{i}+1}}{\left(1+\frac{1}{{℧}_{\alpha_{i},\beta_{i}}}{\left(\frac{{\bar{\gamma }}_{i}}{\gamma_{i}}\right)}^{\beta_{i}}\right)}^{-(\alpha_{i}+1)},$$

(4) $$F_{\gamma_{i}}\left(\gamma_{i}\right)={\left(1+\frac{1}{{℧}_{\alpha_{i},\beta_{i}}}{\left(\frac{{\bar{\gamma }}_{i}}{\gamma_{i}}\right)}^{\beta_{i}}\right)}^{-\alpha_{i}},$$

where the indices i={1;2} denote the subchannels (i.e., “1”—from the transmitter to the IRS and “2”—from the IRS to the receiver), Γ(·) is the Euler gamma function, ${\bar{\gamma }}_{i}$ is the average SNR, and ${℧}_{\alpha_{i},\beta_{i}}$ is defined as

(5) $${℧}_{\alpha_{i},\beta_{i}}={\left(\frac{\Gamma \left(1-\frac{1}{\beta_{i}}\right)\Gamma \left(\alpha_{i}+\frac{1}{\beta_{i}}\right)}{\Gamma (\alpha_{i})}\right)}^{\beta_{i}}.$$

Such a model can handle both fading cases: heavier than Rayleigh (the so-called hyper-Rayleigh regimes) as well as lighter than Rayleigh. Moreover, it can account for heavy fading tails and nonlinear effects of signal propagation.

As further derivations require the IPL envelope statistics, it can be easily obtained via classical random variable transformation, by relating SNR with the envelope [46] (i.e., $|{\dot{h}}_{i}|=\sqrt{\frac{\gamma_{i}}{{\bar{\gamma }}_{i}}\Omega_{i}}$ with $\Omega_{i}=E\{|{\dot{h}}_{i}{|}^{2}\}$), resulting in

(6) $$f_{|{\dot{h}}_{i}|}\left(\right|{\dot{h}}_{i}\left|\right)=2\frac{\alpha_{i}\beta_{i}}{{℧}_{\alpha_{i},\beta_{i}}}\frac{\Omega_{i}^{\beta_{i}}}{|{\dot{h}}_{i}{|}^{2\beta_{i}+1}}{\left(1+\frac{1}{{℧}_{\alpha_{i},\beta_{i}}}{\left(\frac{\Omega_{i}}{|{\dot{h}}_{i}{|}^{2}}\right)}^{\beta_{i}}\right)}^{-(\alpha_{i}+1)}.$$

As the model is completely defined by the parameter set $\left(\alpha_{i},\beta_{i}\right)$, they should be chosen in accordance with the real-life scenarios. For later on, we will use the values extracted from the full-scale measurements performed for two specific cases:

- Higher-frequency (2.4 GHz) long-range (∼1.5 km) cellular communication [47] denoted as “LTE-case” for later on, with $\left(\alpha_{i}=0.59,\beta_{i}=1.77\right)$.
- Lower-frequency (862 MHz) short-range (∼15 m) device-to-device communication [48] denoted as “D2D-case” with $\left(\alpha_{i}=0.45,\beta_{i}=1.55\right)$.

### 2.3. System Performance Metrics

As the primary motivation of the current research is to perform the joint quality–reliability analysis of the assumed IRS-assisted communication system with IPL fading, the quantifying metrics should be introduced. As was proposed in [40], the reliability of the communication link will be described by the outage probability and quality by the average error rate.

#### 2.3.1. Link Reliability Metrics: Outage Probability

The outage probability is commonly defined as the probability that the instantaneous signal-to-noise ratio γ drops below the prespecified threshold value $\gamma_{th}$:

(7) $$P_{out}≜P\left\{\gamma \leq \gamma_{th}\right\}.$$

Thus, by adjusting $\gamma_{th}$, one can control the outage occurrence. On the other hand, it is chosen in such a way to supply the required quality. From the practical perspectives, $P_{out}$ can range from several percent (in high-tolerant applications) up to $10^{-7}÷10^{-8}$ in critical applications (for example, in 6G [3]).

#### 2.3.2. Link Quality Metrics: Average Error Rate

To quantify the performance of the wireless communication system, the research assumes the average bit error rate ${\bar{P}}_{e}$, which is obtained by averaging the SNR-dependent instantaneous BER $P_{e}\left(\gamma_{\Sigma }\right)$ over the channel SNR statistics $f_{\gamma_{\Sigma }}\left(\gamma_{\Sigma }\right)$.

(8) $${\bar{P}}_{er}≜\int_{0}^{\infty }P_{er}\left(\gamma_{\Sigma }\right)f_{\gamma_{\Sigma }}\left(\gamma_{\Sigma }\right)d\gamma$$

- Average bit error rate for coherent modulations.
  For a broad variety of coherent modulations widely used in wireless communications [46] (e.g., BPSK, GMSK (for high $\bar{\gamma }$), M-PSK, square M-QAM, etc.), ABER can be efficiently approximated as a combination of Gauss Q-functions Q(·), i.e.,
  (9) $${\bar{P}}_{er}^{c}=\delta_{1}\sum_{j=1}^{\delta_{3}}\int_{0}^{\infty }Q\left(\sqrt{2\delta_{2,j}\gamma_{\Sigma }}\right)f_{\gamma_{\Sigma }}\left(\gamma_{\Sigma }\right)d\gamma ,$$
  where the combination of the coefficients $\left\{\delta_{1},\delta_{2,j},\delta_{3}\right\}$ is properly defined for the particular modulation. For example, in the case of M-QAM: 41−1Mlog2M,3(2j−1)22(M−1),M2 and for M-PSK: $\left\{\frac{1}{\max(2,\log_{2}M)},2\log_{2}M\sin^{2}\left(\frac{(2j-1)\pi }{M}\right),\max\left(1,\frac{M}{4}\right)\right\}$.
- Average bit error rate for non-coherent modulations.
  For several non-coherent modulations, ABER can be efficiently approximated by the following [46]:
  (10) $${\bar{P}}_{er}^{nc}=\sum_{j=1}^{\varepsilon_{3}}\varepsilon_{1,j}\int_{0}^{\infty }e^{-\varepsilon_{2,j}\gamma_{\Sigma }}f_{\gamma_{\Sigma }}\left(\gamma_{\Sigma }\right)d\gamma_{\Sigma },$$
  where, for example, $\left\{\varepsilon_{1,j},\varepsilon_{2,j},\varepsilon_{3}\right\}$ are equal to (−1)j+1j+1M−1j,jj+1,M−1 for M-FSK and $\left\{\frac{1}{2},1,1\right\}$ for DBPSK.

The solution of the integrals above can be assessed either numerically or analytically (in closed form). The first approach, being more appealing for engineering purposes, faces several major issues with its high sensitivity to the parameter values, problems with the solution’s stability for specific $m,\alpha ,\beta ,\bar{\gamma }$, time-consuming calculations, etc. Thus, in most situations, an analytical solution is sought (if possible).

#### 2.3.3. Joint Quality–Reliability Analysis

Following the premises outlined in the introduction, this study employs a comprehensive evaluation framework that captures the combined impact of the aforementioned metrics (relating to quality and reliability of the system). Specifically, we adopt the joint quality–reliability methodology introduced in [40,49], wherein the link reliability is measured via the outage probability $P_{out}$, while the link quality is determined by the average bit error rate ${\bar{P}}_{er}$. Under this framework, analyzing how the combined $P_{out}-{\bar{P}}_{er}$ characteristic deviates from the bisector line across diverse fading conditions (governed by the transmitter-to-IRS and IRS-to-receiver channels’ configurations) allows us to identify whether the system behavior favors quality or reliability.

## 3. Closed-Form IRS-Assisted Communication System Description

For the system setup adopted in Section 2, the closed-form results regarding the first-order statistical description were presented in [37].

### 3.1. Exact Statistical Analysis

As was pointed out in [37], if the IRS is configured in the far-zone of the transmitter/receiver, the difference of the statistical descriptions between each of the subpaths (i.e., from the transmitter to each of the IRS elements or from each IRS element to the receiver) is negligible; thus, following the framework presented in [37], we will assume that $\forall \;l=1\ldots N_{IRS}$ (where *l* denotes the IRS element): $\alpha_{1,l}=\alpha_{1}$, $\alpha_{2,l}=\alpha_{2}$, $\beta_{1,l}=\beta_{1}$, $\beta_{2,l}=\beta_{2}$. In this case, the closed-form expression for the instantaneous envelope probability density function is given by the following (see Equation (8) [37]):

(11) $$f_{h_{\Sigma }}\left(h_{\Sigma }\right)=\frac{h_{\Sigma }^{-1}}{{\left(\Gamma \left(\alpha_{1}\right)\Gamma \left(\alpha_{2}\right)\right)}^{N_{IRS}}}H_{0,1:\underset{N_{IRS}-times}{\underset{︸}{3,2;\ldots ;3,2}}}^{0,0:\overset{N_{IRS}-times}{\overset{︷}{2,3;\ldots ;2,3}}}\left[\begin{array}{c} \sqrt{\frac{{℧}_{1}^{\frac{1}{\beta_{1}}}{℧}_{2}^{\frac{1}{\beta_{2}}}}{\Omega 1\Omega_{2}}}h_{\Sigma }\\ \vdots \\ \sqrt{\frac{{℧}_{1}^{\frac{1}{\beta_{1}}}{℧}_{2}^{\frac{1}{\beta_{2}}}}{\Omega_{1}\Omega_{2}}}h_{\Sigma } \end{array}\;\;\begin{array}{c} —\\ \vdots \\ \left(1;1\ldots 1\right) \end{array}\begin{array}{c} :\\ : \end{array}\begin{array}{c} \left(0,\frac{1}{2\beta_{1}}\right);\ldots ;\left(0,\frac{1}{2\beta_{1}}\right);\left(0,\frac{1}{2\beta_{2}}\right);\ldots ;\left(0,\frac{1}{2\beta_{2}}\right);\left(1,1\right)\\ \vdots \\ \left(\alpha_{1},\frac{1}{2\beta_{1}}\right);\ldots ;\left(\alpha_{1},\frac{1}{2\beta_{1}}\right);\left(\alpha_{2},\frac{1}{2\beta_{2}}\right);\ldots ;\left(\alpha_{2},\frac{1}{2\beta_{2}}\right) \end{array}\right],$$

where H(·) is the multivariate Fox H-function (see Appendix A.1 in [50]). It must be specifically pointed out that initially in [37], the solution is given in terms of a multivariate integral; thus, it converges and uniquely represents Fox H-function (11) only if the proper integration contour is chosen, i.e., it should be chosen in such a way to separate the positive and negative poles of the integrands (that is, gamma functions) of the numerator. For example, for the *l*-th integration variable $\sigma_{l}$, the contour $H_{\sigma_{l}}:\left(ℜ\left\{\sigma_{l}\right\}-i\infty ;ℜ\left\{\sigma_{l}\right\}+i\infty \right)$ with $0<ℜ\left\{\sigma_{l}\right\}<2\min\left\{\alpha_{1}\beta_{1};\alpha_{2}\beta_{2}\right\}$

Although (11) gives an exact solution for the envelope at the receiver, the expression is too complex for further derivations and has multiple issues with direct implementation. Thus, for the subsequent analysis we will resort to the proposed approximation.

### 3.2. Approximate Analysis

As all the primary metrics under investigation are given in terms of the instantaneous SNR rather than channel coefficient, we will use the SNR PDF $f_{\gamma_{\Sigma }}\left(\gamma_{\Sigma }\right)$ approximation given in [37] (see Theorem 3):

(12) $$f_{\gamma_{\Sigma }}\left(\gamma_{\Sigma }\right)=\frac{1}{2\gamma_{\Sigma }\Gamma \left(\alpha_{g}\right)}{\left(\frac{\alpha_{g}\left(\alpha_{g}+1\right)\gamma_{\Sigma }}{{\bar{\gamma }}_{\Sigma }}\right)}^{\frac{\alpha_{g}}{2}-1}e^{-\sqrt{\frac{\alpha_{g}\left(\alpha_{g}+1\right)\gamma_{\Sigma }}{{\bar{\gamma }}_{\Sigma }}}},$$

where ${\bar{\gamma }}_{\Sigma }$ is the average SNR and the approximation coefficient $\alpha_{g}$ is explicitly given by

(13) $$\alpha_{g}=\frac{N_{IRS}}{\frac{\Gamma \left(\alpha_{1}+\frac{1}{\beta_{1}}\right)\Gamma \left(1-\frac{1}{\beta_{1}}\right)\Gamma \left(\alpha_{2}+\frac{1}{\beta_{2}}\right)\Gamma \left(1-\frac{1}{\beta_{2}}\right)\Gamma \left(\alpha_{1}\right)\Gamma \left(\alpha_{2}\right)}{{\left(\Gamma \left(\alpha_{1}+\frac{1}{2\beta_{1}}\right)\Gamma \left(1-\frac{1}{2\beta_{1}}\right)\Gamma \left(\alpha_{2}+\frac{1}{2\beta_{2}}\right)\Gamma \left(1-\frac{1}{2\beta_{2}}\right)\right)}^{2}}-1}.$$

In this case, the cumulative distribution function of the instantaneous SNR at the receiver can be easily obtained as follows:

(14) $$F_{\gamma_{\Sigma }}\left(\gamma_{\Sigma }\right)=1-\frac{\Gamma \left(\alpha_{g},\sqrt{\frac{\alpha_{g}\left(\alpha_{g}+1\right)\gamma_{\Sigma }}{{\bar{\gamma }}_{\Sigma }}}\right)}{\Gamma \left(\alpha_{g}\right)}.$$

It can be noted that the channel parameters ($\alpha_{1},\alpha_{2},\beta_{1},\beta_{2}$) as well as the IRS size (i.e., $N_{IRS}$) have a nonlinear cumulative impact on the approximation parameter $\alpha_{g}$, which controls the first-order statistical description.

## 4. IRS-Assisted Communication System Performance: Derived Analytical Results

To derive the closed-form expression for the JQR analysis, one needs to link the outage probability with the average error rate metric. This can be achieved by noting that they are evaluated for a given average SNR, which states the connection between the two. As the reliability metric is explicitly represented in terms of the obtained earlier CDF (see (7)), in the first step, it is easier to evaluate ${\bar{\gamma }}_{\Sigma }$ in terms of $P_{out}$ analytically (if virtually possible) and, afterwards, substitute it into the derived expression for ${\bar{P}}_{er}$.

### 4.1. Outage Probability

Employing the definition of the outage probability (see (7)) and combining it with the expression for the CDF (14)):

(15) $$P_{out}=F_{\gamma_{\Sigma }}\left(\gamma_{\Sigma_{th}}\right)=1-\frac{\Gamma \left(\alpha_{g},\sqrt{\frac{\alpha_{g}\left(\alpha_{g}+1\right)\gamma_{\Sigma_{th}}}{{\bar{\gamma }}_{\Sigma }}}\right)}{\Gamma \left(\alpha_{g}\right)},$$

where $\gamma_{\Sigma_{th}}$ is the threshold SNR defining the outage event. Hence, equating the average SNR $\gamma_{\Sigma }$, yields

(16) $${\bar{\gamma }}_{\Sigma }=\frac{\alpha_{g}\left(\alpha_{g}+1\right)\gamma_{\Sigma_{th}}}{{\left(\Gamma^{-1}\left(\alpha_{g},1-P_{out}\right)\right)}^{2}},$$

where $\Gamma^{-1}\left(a,z\right)$ is the inverse incomplete upper gamma-function [51], i.e., the solution *s* of the equation $z=\frac{\Gamma (a,s)}{\Gamma (a)}$.

### 4.2. Average Bit Error Rate

Having at hand the expression of ${\bar{\gamma }}_{\Sigma }$ as a function of $P_{out}$, one needs to derive the closed-form representation for the assumed quality metrics.

**Theorem** **1.** *The closed-form expressions for the average error rate of the IRS-assisted communication system with IPL fading in each segment for non-coherent ${\bar{P}}_{er}^{nc}$ and coherent ${\bar{P}}_{er}^{c}$ modulations are given by*

(17) $${\bar{P}}_{er}^{nc}={\left(\frac{\alpha_{g}\left(\alpha_{g}+1\right)}{4{\bar{\gamma }}_{\Sigma }}\right)}^{\frac{\alpha_{g}}{2}}\sum_{j=1}^{\varepsilon_{3}}\varepsilon_{1,j}\varepsilon_{2,j}^{-\frac{\alpha_{g}}{2}}U\left(\frac{\alpha_{g}}{2};\frac{1}{2};\frac{\alpha_{g}\left(\alpha_{g}+1\right)}{4{\bar{\gamma }}_{\Sigma }\varepsilon_{2,j}}\right),$$

(18) $${\bar{P}}_{er}^{c}=\frac{\delta_{1}2^{\alpha_{g}-2}}{\pi \Gamma (\alpha_{g})}\sum_{j=1}^{\delta_{3}}G_{2,3}^{2,2}\left(\frac{\alpha_{g}\left(\alpha_{g}+1\right)}{4{\bar{\gamma }}_{\Sigma }\delta_{2,j}}|\begin{array}{c} \frac{1}{2},1\\ \frac{\alpha_{g}}{2},\frac{\alpha_{g}+1}{2},0 \end{array}\right),$$

*where $G_{2,3}^{2,2}\left(\cdot \right)$ is the Meijer G-function (Section 13.17 [51]) and U(·) is the Tricomi’s (confluent hypergeometric) function (Chapter 13 [51])*.

**Proof.**  To start the proof, it should be noted that the expressions for the ABER of coherent and non-coherent modulations can be expressed as the Laplace transform (i.e., $L\left\{\cdot ;x\right\}$ evaluated at *x*) of the scaled CDF $F_{\gamma_{\Sigma }}\left(\gamma_{\Sigma }\right)$ of the instantaneous SNR as follows (for details see [29]):

(19) $${\bar{P}}_{er}^{c}=\frac{\delta_{1}}{2\sqrt{\pi }}\sum_{j=1}^{\delta_{3}}\sqrt{\delta_{2,j}}L\left\{\frac{F_{\gamma_{\Sigma }}\left(\gamma_{\Sigma }\right)}{\sqrt{\gamma_{\Sigma }}};\delta_{2,j}\right\},$$

(20) $${\bar{P}}_{er}^{nc}=\sum_{j=1}^{\varepsilon_{3}}\varepsilon_{1,j}\varepsilon_{2,j}L\left\{F_{\gamma_{\Sigma }}\left(\gamma_{\Sigma }\right);\varepsilon_{2,j}\right\}.$$

Express $F_{\gamma_{\Sigma }}\left(\gamma_{\Sigma }\right)$ in terms of Meijer G-function via

(21) $$F_{\gamma_{\Sigma }}\left(\gamma_{\Sigma }\right)=\frac{2^{\alpha_{g}-1}}{\sqrt{\pi }\Gamma \left(\alpha_{g}\right)}G_{1,3}^{2,1}\left(\frac{\alpha_{g}\left(\alpha_{g}+1\right)\gamma_{\Sigma }}{4{\bar{\gamma }}_{\Sigma }}|\begin{array}{c} 1\\ \frac{\alpha_{g}}{2},\frac{\alpha_{g}+1}{2},0 \end{array}\right).$$

Thus, the ABER for non-coherent modulations can be directly obtained by the Laplace transform of (21) (see Equation 3.40.1 [52]):

(22) $$L\left\{G_{1,3}^{2,1}\left(\frac{\alpha_{g}\left(\alpha_{g}+1\right)\gamma_{\Sigma }}{4{\bar{\gamma }}_{\Sigma }}|\begin{array}{c} 1\\ \frac{\alpha_{g}}{2},\frac{\alpha_{g}+1}{2},0 \end{array}\right);\varepsilon_{2,j}\right\}=\frac{1}{\varepsilon_{2,j}}G_{2,3}^{2,2}\left(\frac{\alpha_{g}\left(\alpha_{g}+1\right)}{4{\bar{\gamma }}_{\Sigma }\varepsilon_{2,j}}|\begin{array}{c} 0,1\\ \frac{\alpha_{g}}{2},\frac{\alpha_{g}+1}{2},0 \end{array}\right).$$

The obtained expression can be further simplified by utilizing the Mellin–Barnes contour integral representation of the Meijer G-function; after simplifying the integrands, the expression reduces to the Mellin–Barnes representation (see Equation 13.4.17 [51]) of the Tricomi’s (confluent hypergeometric) function (Chapter 13 [51]):

(23) $$G_{2,3}^{2,2}\left(\frac{\alpha_{g}\left(\alpha_{g}+1\right)}{4{\bar{\gamma }}_{\Sigma }\varepsilon_{2,j}}|\begin{array}{c} 0,1\\ \frac{\alpha_{g}}{2},\frac{\alpha_{g}+1}{2},0 \end{array}\right)={\left(\frac{\alpha_{g}\left(\alpha_{g}+1\right)}{4{\bar{\gamma }}_{\Sigma }\varepsilon_{2,j}}\right)}^{\frac{\alpha_{g}}{2}}\Gamma \left(\frac{\alpha_{g}}{2}\right)\Gamma \left(\frac{\alpha_{g}+1}{2}\right)U\left(\frac{\alpha_{g}}{2};\frac{1}{2};\frac{\alpha_{g}\left(\alpha_{g}+1\right)}{4{\bar{\gamma }}_{\Sigma }\varepsilon_{2,j}}\right),$$

where U(·) is the Tricomi’s (confluent hypergeometric) function (Chapter 13 [51]). After some direct simplifications, the expression (17) follows.

To prove (18), one can use the same approach, i.e., express the instantaneous SNR CDF in terms of Meijer G-function and apply the Laplace transform (via Equation 3.40.1 [52]):

(24) $$L\left\{\gamma_{\Sigma }^{-\frac{1}{2}}G_{1,3}^{2,1}\left(\frac{\alpha_{g}\left(\alpha_{g}+1\right)\gamma_{\Sigma }}{4{\bar{\gamma }}_{\Sigma }}|\begin{array}{c} 1\\ \frac{\alpha_{g}}{2},\frac{\alpha_{g}+1}{2},0 \end{array}\right);\delta_{2,j}\right\}=\frac{1}{\sqrt{\delta_{2,j}}}G_{2,3}^{2,2}\left(\frac{\alpha_{g}\left(\alpha_{g}+1\right)}{4{\bar{\gamma }}_{\Sigma }\delta_{2,j}}|\begin{array}{c} \frac{1}{2},1\\ \frac{\alpha_{g}}{2},\frac{\alpha_{g}+1}{2},0 \end{array}\right).$$

Simplifying the terms and reducing the summands yields

(25) $${\bar{P}}_{er}^{c}=\frac{\delta_{1}2^{\alpha_{g}-2}}{\pi \Gamma (\alpha_{g})}\sum_{j=1}^{\delta_{3}}G_{2,3}^{2,2}\left(\frac{\alpha_{g}\left(\alpha_{g}+1\right)}{4{\bar{\gamma }}_{\Sigma }\delta_{2,j}}|\begin{array}{c} \frac{1}{2},1\\ \frac{\alpha_{g}}{2},\frac{\alpha_{g}+1}{2},0 \end{array}\right),$$

which finalizes the proof of Theorem 1. □

It should be noted that the expressions derived are presented in terms of functions implemented in most computer algebra systems (e.g., MatLAB (starting from version R2017b), Mathematica (starting form v3.0), Maple (starting from release v5), etc.) or that can be assessed via computational routines available in most computer languages (e.g., C, C♯, Python, etc.). Nevertheless, if required, the obtained solutions for the ABER can be easily evaluated via the numerical integration of their Mellin–Barnes representation. For example, for the last expression (i.e., ABER for coherent modulations):

(26) $$\begin{array}{cc} G_{2,3}^{2,2}\left(\frac{\alpha_{g}\left(\alpha_{g}+1\right)}{4{\bar{\gamma }}_{\Sigma }\delta_{2,j}}|\begin{array}{c} \frac{1}{2},1\\ \frac{\alpha_{g}}{2},\frac{\alpha_{g}+1}{2},0 \end{array}\right) & =\\ \; & \;=\frac{1}{2\pi i}\int_{H}\frac{\Gamma \left(\frac{\alpha_{g}}{2}+\sigma \right)\Gamma \left(\frac{\alpha_{g}+1}{2}+\sigma \right)\Gamma \left(\frac{1}{2}-\sigma \right)\Gamma \left(-\sigma \right)}{\Gamma (1-\sigma )}{\left(\frac{\alpha_{g}\left(\alpha_{g}+1\right)}{4{\bar{\gamma }}_{\Sigma }\delta_{2,j}}\right)}^{-\sigma }d\sigma , \end{array}$$

where the contour $H:(-a^{*}-i\infty ,-a^{*}+i\infty )$ and $-\frac{\alpha_{g}}{2}<a^{*}<0$. Or, alternatively, by noting that $\frac{\Gamma (-\sigma )}{\Gamma (1-\sigma )}=-\frac{1}{\sigma }$, the contour integral can be simplified even further, as follows:

(27) $$\begin{array}{cc} G_{2,3}^{2,2}\left(\frac{\alpha_{g}\left(\alpha_{g}+1\right)}{4{\bar{\gamma }}_{\Sigma }\delta_{2,j}}|\begin{array}{c} \frac{1}{2},1\\ \frac{\alpha_{g}}{2},\frac{\alpha_{g}+1}{2},0 \end{array}\right) & =\\ \; & \;=-\frac{1}{2\pi i}\int_{H}\frac{\Gamma \left(\frac{\alpha_{g}}{2}+\sigma \right)\Gamma \left(\frac{\alpha_{g}+1}{2}+\sigma \right)\Gamma \left(\frac{1}{2}-\sigma \right)}{\sigma }{\left(\frac{\alpha_{g}\left(\alpha_{g}+1\right)}{4{\bar{\gamma }}_{\Sigma }\delta_{2,j}}\right)}^{-\sigma }d\sigma , \end{array}$$

with the same integration contour H.

For practical applications, the derived expressions for the average error rates must be supplied with their asymptotic behavior. As the system performance is governed by average SNR and number of IRS elements, both their asymptotics are required.

### 4.3. High-SNR Asymptotic Analysis

**Theorem** **2.** *The high-SNR approximation of the average error rate of the IRS-assisted communication system with IPL fading in each segment for non-coherent ${\left.{\bar{P}}_{er}^{c}\right|}_{{\bar{\gamma }}_{\Sigma }\to \infty }$ and coherent ${\left.{\bar{P}}_{er}^{nc}\right|}_{{\bar{\gamma }}_{\Sigma }\to \infty }$ modulations are given by*

(28) $${\left.{\bar{P}}_{er}^{nc}\right|}_{{\bar{\gamma }}_{\Sigma }\to \infty }≃\frac{\sqrt{\pi }}{\Gamma \left(\frac{\alpha_{g}+1}{2}\right)}{\left(\frac{\alpha_{g}\left(\alpha_{g}+1\right)}{4{\bar{\gamma }}_{\Sigma }}\right)}^{\frac{\alpha_{g}}{2}}\sum_{j=1}^{\varepsilon_{3}}\varepsilon_{1,j}\varepsilon_{2,j}^{-\frac{\alpha_{g}}{2}},$$

(29) $${\left.{\bar{P}}_{er}^{c}\right|}_{{\bar{\gamma }}_{\Sigma }\to \infty }≃\frac{\delta_{1}}{2\sqrt{\pi }}\frac{\Gamma \left(\frac{\alpha_{g}+1}{2}\right)}{\Gamma (\alpha_{g}+1)}{\left(\frac{\alpha_{g}\left(\alpha_{g}+1\right)}{4{\bar{\gamma }}_{\Sigma }}\right)}^{\frac{\alpha_{g}}{2}}\sum_{j=1}^{\delta_{3}}\delta_{2,j}^{-\frac{\alpha_{g}}{2}},$$

**Proof.**  To prove (28), one can use the asymptotic expansion of the Tricomi function (see Equation 13.2.20 in [51]):

(30) $${\left.U\left(\frac{\alpha_{g}}{2};\frac{1}{2};\frac{\alpha_{g}\left(\alpha_{g}+1\right)}{4{\bar{\gamma }}_{\Sigma }\varepsilon_{2,j}}\right)\right|}_{{\bar{\gamma }}_{\Sigma }\to \infty }≃\frac{\sqrt{\pi }}{\Gamma \left(\frac{\alpha_{g}+1}{2}\right)}.$$

The asymptotic expression for the ${\bar{P}}_{er}^{c}$ can be obtained by noting that Meijer G-function $G_{2,3}^{2,2}\left(\cdot \right)$ can be equivalently represented as Fox H-function $H_{2,3}^{2,2}\left(\cdot \right)$ and applying to the latter the dominant term polynomial-type expansion (see Corollary 1.11.1 in [53]):

(31) $${\left.G_{2,3}^{2,2}\left(\frac{\alpha_{g}\left(\alpha_{g}+1\right)}{4{\bar{\gamma }}_{\Sigma }\delta_{2,j}}|\begin{array}{c} \frac{1}{2},1\\ \frac{\alpha_{g}}{2},\frac{\alpha_{g}+1}{2},0 \end{array}\right)\right|}_{{\bar{\gamma }}_{\Sigma }\to \infty }≃\frac{2\sqrt{\pi }\Gamma \left(\frac{\alpha_{g}+1}{2}\right)}{\Gamma (\alpha_{g})}{\left(\frac{\alpha_{g}\left(\alpha_{g}+1\right)}{4{\bar{\gamma }}_{\Sigma }\delta_{2,j}}\right)}^{\frac{\alpha_{g}}{2}}.$$

□

More importantly, the derived results allow us to derive the large-$N_{IRS}$ asymptotics with the significant consequences, which follow.

### 4.4. Large-IRS Analysis

**Theorem** **3.** *Large-scale IRS-assisted wireless communication systems achieve the fading-free (i.e., flat stationary non-fading channel with AWGN at the receiver) average error rate with the average SNR penalty factor of $\frac{\alpha_{g}}{\alpha_{g}+1}$, irrespective of modulation type (e.g., coherent or non-coherent), i.e., ${\left.{\bar{P}}_{er}\right|}_{N_{IRS}\to \infty }≃P_{er}\left(\frac{\alpha_{g}}{\alpha_{g}+1}{\bar{\gamma }}_{\Sigma }\right)$*.

**Proof.**  As the theorem claims that the results equally hold both for coherent and non-coherent modulations, first let us prove it for the coherent one.

Assume the following set of transformations:

(32a) $$\begin{array}{cccc} & {\left.{\bar{P}}_{er}^{c}\right|}_{N_{IRS}\to \infty } & = & {\left.\frac{\delta_{1}2^{\alpha_{g}-2}}{\pi \Gamma (\alpha_{g})}\sum_{j=1}^{\delta_{3}}G_{2,3}^{2,2}\left(\frac{\alpha_{g}\left(\alpha_{g}+1\right)}{4{\bar{\gamma }}_{\Sigma }\delta_{2,j}}|\begin{array}{c} \frac{1}{2},1\\ \frac{\alpha_{g}}{2},\frac{\alpha_{g}+1}{2},0 \end{array}\right)\right|}_{N_{IRS}\to \infty }=\\ & & & \;=\frac{\delta_{1}}{4\pi }\sum_{j=1}^{\delta_{3}}\frac{1}{2\pi i}\int_{H}\frac{\Gamma \left(\frac{1}{2}-\sigma \right)\Gamma \left(-\sigma \right)}{\Gamma (1-\sigma )}{\left(\frac{\alpha_{g}\left(\alpha_{g}+1\right)}{4{\bar{\gamma }}_{\Sigma }\delta_{2,j}}\right)}^{-\sigma }\times \end{array}\;\;\;\;\;$$

(32b) $$\begin{array}{cccc} & & & \;\times {\left.\left(\frac{\Gamma \left(\frac{\alpha_{g}}{2}+\sigma \right)\Gamma \left(\frac{\alpha_{g}+1}{2}+\sigma \right)}{2^{-\alpha_{g}}\Gamma \left(\alpha_{g}\right)}\right)\right|}_{N_{IRS}\to \infty }d\sigma ≃ \end{array}$$

(32c) $$\begin{array}{cccc} & & ≃ & \frac{\delta_{1}}{2\sqrt{\pi }}\sum_{j=1}^{\delta_{3}}\frac{1}{2\pi i}\int_{H}\frac{\Gamma \left(\frac{1}{2}-\sigma \right)\Gamma \left(-\sigma \right)}{\Gamma (1-\sigma )}{\left(\frac{\left(\alpha_{g}+1\right)}{\alpha_{g}{\bar{\gamma }}_{\Sigma }\delta_{2,j}}\right)}^{-\sigma }d\sigma = \end{array}$$

(32d) $$\begin{array}{cccc} & & ≃ & \frac{\delta_{1}}{2\sqrt{\pi }}\sum_{j=1}^{\delta_{3}}G_{2,1}^{0,2}\left(\frac{\left(\alpha_{g}+1\right)}{\alpha_{g}{\bar{\gamma }}_{\Sigma }\delta_{2,j}}|\begin{array}{c} \frac{1}{2},1\\ 0 \end{array}\right)= \end{array}\;\;\;\;\;\;\;\;$$

(32e) $$\begin{array}{cccc} & & = & \;\;\delta_{1}\sum_{j=1}^{\delta_{3}}Q\left(\sqrt{\frac{\alpha_{g}{\bar{\gamma }}_{\Sigma }\delta_{2,j}}{\left(\alpha_{g}+1\right)}}\right)= \end{array}\;\;\;\;\;\;\;\;\;\;\;$$

(32f) $$\begin{array}{cccc} & & = & \;\;P_{er}^{c}\left(\frac{\alpha_{g}{\bar{\gamma }}_{\Sigma }}{\left(\alpha_{g}+1\right)}\right). \end{array}\;\;\;\;\;\;\;\;\;\;\;\;\;\;$$

The expression (32b) is the Mellin–Barnes representation of (32a); the (32c) was derived from (32b) by taking the limit $N_{IRS}\to \infty$ and noting that this is equivalent to $\alpha_{g}\to \infty$ (as $\alpha_{g}$ linearly scales with $N_{IRS}$):

(33) $${\left.\left(\frac{\Gamma \left(\frac{\alpha_{g}}{2}+\sigma \right)\Gamma \left(\frac{\alpha_{g}+1}{2}+\sigma \right)}{2^{-\alpha_{g}}\Gamma \left(\alpha_{g}\right)}\right)\right|}_{\alpha_{g}\to \infty }≃2\sqrt{\pi }{\left(\frac{4}{\alpha_{g}^{2}}\right)}^{-\sigma }.$$

Noting that the obtained contour integral in (32d) is a simplified Meijer G-function, relating it to the Gaussian Q-function via

(34) $$G_{2,1}^{0,2}\left(\frac{\left(\alpha_{g}+1\right)}{\alpha_{g}{\bar{\gamma }}_{\Sigma }\delta_{2,j}}|\begin{array}{c} \frac{1}{2},1\\ 0 \end{array}\right)=\sqrt{\pi }\left(1-\mathrm{erf}\left(\sqrt{\frac{\alpha_{g}{\bar{\gamma }}_{\Sigma }\delta_{2,j}}{\left(\alpha_{g}+1\right)}}\right)\right)=2Q\left(\sqrt{\frac{\alpha_{g}{\bar{\gamma }}_{\Sigma }\delta_{2,j}}{\left(\alpha_{g}+1\right)}}\right),$$

and by matching (32e) with the definition of the error rate of the coherent modulations with non-fading channel in the presence of the additive white Gaussian noise, one finalizes the first part of the proof.

Performing the same set of transformations (i.e., Mellin–Barnes representation, limiting operation with $\alpha_{g}\to \infty$ and relating to the error rate expression for the non-fading case) for the non-coherent modulations, yields

(35a) $$\begin{array}{cccc} & {\left.{\bar{P}}_{er}^{nc}\right|}_{N_{IRS}\to \infty } & = & {\left.{\left(\frac{\alpha_{g}\left(\alpha_{g}+1\right)}{4{\bar{\gamma }}_{\Sigma }}\right)}^{\frac{\alpha_{g}}{2}}\sum_{j=1}^{\varepsilon_{3}}\varepsilon_{1,j}\varepsilon_{2,j}^{-\frac{\alpha_{g}}{2}}U\left(\frac{\alpha_{g}}{2};\frac{1}{2};\frac{\alpha_{g}\left(\alpha_{g}+1\right)}{4{\bar{\gamma }}_{\Sigma }\varepsilon_{2,j}}\right)\right|}_{N_{IRS}\to \infty }=\\ & & & \;={\left(\frac{\alpha_{g}\left(\alpha_{g}+1\right)}{4{\bar{\gamma }}_{\Sigma }}\right)}^{\frac{\alpha_{g}}{2}}\sum_{j=1}^{\varepsilon_{3}}\varepsilon_{1,j}\varepsilon_{2,j}^{-\frac{\alpha_{g}}{2}}\frac{1}{2\pi i}\int_{-i\infty }^{i\infty }\Gamma \left(-\sigma \right){\left(\frac{\alpha_{g}\left(\alpha_{g}+1\right)}{4{\bar{\gamma }}_{\Sigma }\varepsilon_{2,j}}\right)}^{-\sigma -\frac{\alpha_{g}}{2}}\times \end{array}$$

(35b) $$\begin{array}{cccc} & & & \;\times {\left.\left(\frac{\Gamma \left(\frac{\alpha_{g}}{2}+\sigma \right)\Gamma \left(\frac{\alpha_{g}+1}{2}+\sigma \right)}{\Gamma \left(\frac{\alpha_{g}}{2}\right)\Gamma \left(\frac{\alpha_{g}+1}{2}\right)}\right)\right|}_{N_{IRS}\to \infty }d\sigma ≃ \end{array}$$

(35c) $$\begin{array}{cccc} & & ≃ & \sum_{j=1}^{\varepsilon_{3}}\varepsilon_{1,j}\frac{1}{2\pi i}\int_{-i\infty }^{i\infty }\Gamma \left(-\sigma \right){\left(\frac{\left(\alpha_{g}+1\right)}{\alpha_{g}{\bar{\gamma }}_{\Sigma }\varepsilon_{2,j}}\right)}^{-\sigma }d\sigma = \end{array}$$

(35d) $$\begin{array}{cccc} & & ≃ & \sum_{j=1}^{\varepsilon_{3}}\varepsilon_{1,j}e^{-\varepsilon_{2,j}\left(\frac{\alpha_{g}{\bar{\gamma }}_{\Sigma }}{\left(\alpha_{g}+1\right)}\right)}= \end{array}\;\;\;\;\;\;\;$$

(35e) $$\begin{array}{cccc} & & = & \;\;P_{er}^{nc}\left(\frac{\alpha_{g}{\bar{\gamma }}_{\Sigma }}{\left(\alpha_{g}+1\right)}\right). \end{array}\;\;\;\;\;\;\;\;\;$$

Here, (35b) is obtained as the first-order terms of Taylor expansion of the ratio of gamma functions:

(36) $${\left.\left(\frac{\Gamma \left(\frac{\alpha_{g}}{2}+\sigma \right)\Gamma \left(\frac{\alpha_{g}+1}{2}+\sigma \right)}{\Gamma \left(\frac{\alpha_{g}}{2}\right)\Gamma \left(\frac{\alpha_{g}+1}{2}\right)}\right)\right|}_{\alpha_{g}\to \infty }≃{\left(\frac{4}{\alpha_{g}^{2}}\right)}^{-\sigma },$$

and (35c) is evaluated via the well-known Cahen–Mellin integral (see Equation 1.11 in [54]):

(37) $$\frac{1}{2\pi i}\int_{\chi -i\infty }^{\chi +i\infty }\Gamma \left(\sigma \right)z^{-\sigma }d\sigma =e^{-z}.$$

This finalizes the proof of Theorem 3. □

The latter result has a very important theoretical consequence formulated in the following Corollary.

**Corollary** **1.** *In the limiting case (i.e., extremely large IRS) of $N_{IRS}\to \infty$ the IRS completely eliminates the effects of fading on link quality quantified in terms of the error rate*.

**Proof.**  The Corollary trivially follows from the fact that

(38) $$\lim_{N_{IRS}\to \infty }\frac{\alpha_{g}}{\alpha_{g}+1}=\lim_{\alpha_{g}\to \infty }\frac{\alpha_{g}}{\alpha_{g}+1}=1.$$

□

Thus, from Corollary 1 it is clear that the fading channel parameters (which govern the expression for $\alpha_{g}$) act as a penalty factor for the average SNR at the receiver. Moreover, the presented expressions help to identify the number of IRS elements needed to control this penalty on a reasonable level, thus giving a solid ground for the system designers for further IRS synthesis and optimization.

### 4.5. Joint Q–R Analysis

Finally, the obtained results allow us to derive JQR analysis (i.e., the Q–R curve expression).

**Theorem** **4.** *The unified expression of the Q–R curve for arbitrary modulation type (i.e., coherent or non-coherent) can be expressed in the following form:*

(39) $${\bar{P}}_{er}=\frac{2^{\alpha_{g}-1}}{\sqrt{\pi }\Gamma \left(\alpha_{g}\right)}\sum_{j=1}^{\xi_{3}}\xi_{1,j}G_{\xi_{4}+1,\xi_{4}+2}^{2,\xi_{4}+1}\left(\frac{{\left(\Gamma^{-1}\left(\alpha_{g},1-P_{out}\right)\right)}^{2}}{4\gamma_{\Sigma_{th}}\xi_{2,j}}|\begin{array}{c} 1,\xi_{5}\\ \frac{\alpha_{g}}{2},\frac{\alpha_{g}+1}{2},\xi_{6} \end{array}\right),$$

*where the modulation-dependent coefficients ξ are defined as follows (the upper cases always correspond to non-coherent modulation, whereas the lower cases for coherent):*

$$\xi_{1,j}=\left\{\begin{array}{cc} \varepsilon_{1,j}, & \\ \frac{\delta_{1,j}}{2\sqrt{\pi }}, \end{array}\right.\;\;\xi_{2,j}=\left\{\begin{array}{cc} \varepsilon_{2,j}, & \\ \delta_{2,j}, \end{array}\right.\;\;\xi_{3}=\left\{\begin{array}{cc} \varepsilon_{3}, & \\ \delta_{3}, \end{array}\right.\;\;\xi_{4}=\left\{\begin{array}{cc} 0, & \\ 1, \end{array}\right.\;\;\xi_{5}=\left\{\begin{array}{cc} –, & \\ \frac{1}{2}, \end{array}\right.\;\;\xi_{6}=\left\{\begin{array}{cc} –, & \\ 0, \end{array}\right.$$

**Proof.**  To prove Theorem 4, one can generalize the expression for the Tricomi function (the case of non-coherent modulations) in the following way:

(40) $$\;{\left(\frac{\alpha_{g}\left(\alpha_{g}+1\right)}{4{\bar{\gamma }}_{\Sigma }\varepsilon_{2,j}}\right)}^{\frac{\alpha_{g}}{2}}U\left(\frac{\alpha_{g}}{2};\frac{1}{2};\frac{\alpha_{g}\left(\alpha_{g}+1\right)}{4{\bar{\gamma }}_{\Sigma }\varepsilon_{2,j}}\right)=\frac{{\left(\frac{\alpha_{g}\left(\alpha_{g}+1\right)}{4{\bar{\gamma }}_{\Sigma }\varepsilon_{2,j}}\right)}^{\frac{\alpha_{g}}{2}}G_{1,2}^{2,1}\left(\frac{\alpha_{g}\left(\alpha_{g}+1\right)}{4{\bar{\gamma }}_{\Sigma }\varepsilon_{2,j}}|\begin{array}{c} 1\\ \frac{\alpha_{g}}{2},\frac{\alpha_{g}+1}{2} \end{array}\right)}{\Gamma \left(\frac{\alpha_{g}}{2}\right)\Gamma \left(\frac{\alpha_{g}+1}{2}\right)}.$$

Next, apply the power property of Meijer G-functions (see Equation (16.19.2) in [51]) and use the fact that

(41) $$\frac{1}{\Gamma \left(\frac{\alpha_{g}}{2}\right)\Gamma \left(\frac{\alpha_{g}+1}{2}\right)}=\frac{2^{\alpha_{g}-1}}{\sqrt{\pi }\Gamma \left(\alpha_{g}\right)}.$$

Matching the obtained expression with the result for the ABER for coherent modulations (18) and substituting the average SNR expressed as a function of the outage probability finalizes the proof. □

The obtained results can be further analyzed in asymptotic forms (for both high-SNR and large-$N_{IRS}$ asymptotics), which helps to provide a better insight into the state of possible improvements due to hardware modifications or propagation conditions.

**Theorem** **5.** *The unified asymptotic expression of the Q–R curve for arbitrary modulation type (i.e., coherent or non-coherent) can be expressed in the following form:*

- *For the case of high-SNR asymptotics:*
(42) $${\bar{P}}_{er}={\left(\frac{\Gamma^{-1}\left(\alpha_{g},1-P_{out}\right)}{2}\right)}^{\alpha_{g}}\xi_{7}\sum_{j=1}^{\xi_{3}}\xi_{1,j}\xi_{2,j}^{-\frac{\alpha_{g}}{2}},\;\;\xi_{7}=\left\{\begin{array}{cc} \frac{\sqrt{\pi }}{\Gamma \left(\frac{\alpha_{g}+1}{2}\right)},\;\;\;\mathrm{non}\text{-}\mathrm{coherent} & \\ \frac{\sqrt{\pi }2^{-\alpha_{g}}}{\Gamma \left(\frac{\alpha_{g}}{2}+1\right)},\;\;\;\mathrm{coherent} \end{array}\right.$$
- *For the case large-scale IRS asymptotics:*
(43) $${\left.{\bar{P}}_{er}\right|}_{N_{IRS}\to \infty }≃P_{er}\left({\left(\frac{\alpha_{g}}{\Gamma^{-1}\left(\alpha_{g},1-P_{out}\right)}\right)}^{2}\gamma_{\Sigma_{th}}\right)$$

**Proof.**  After the introduction of the modulation-dependent coefficient $\xi_{7}$, the proof trivially follows from the sequential application of the results presented in Theorems 2 and 3. □

## 5. System Performance: Numerical Analysis

To verify the performed analytical work, establish the correctness of the derived expressions, and perform the joint quality–reliability analysis, a numeric simulation was carried out.

The overall simulation was performed in full alignment with the general theoretical frameworks presented in Section 2 (see Section 2.1 for the IRS-assisted system model description, Section 2.2 for the statistical channel model under consideration, and Section 2.3 for the performance metrics under investigation).

The system setup and channel parameters with their respective values are listed in Table 1.

To this extent, several important notes should be pointed out:

- The IPL fading channel parameters were extracted from the experimental verification of the IPL model (as was shown earlier) and correspond to the hyper-Rayleigh behavior of the propagation channel.
- All the fading channels between the source and each element of the IRS were assumed statistically equivalent (i.e., the statistical channel parameters were set to be equal); the same was performed with the channels between the IRS and the destination point.
- The number of IRS elements $N_{IRS}$ was restricted to 64 (not a very large panel) as it was demonstrated in [37] that for such a channel model even a moderate number of elements was sufficient to yield sufficient improvements.

**Figure 1:**
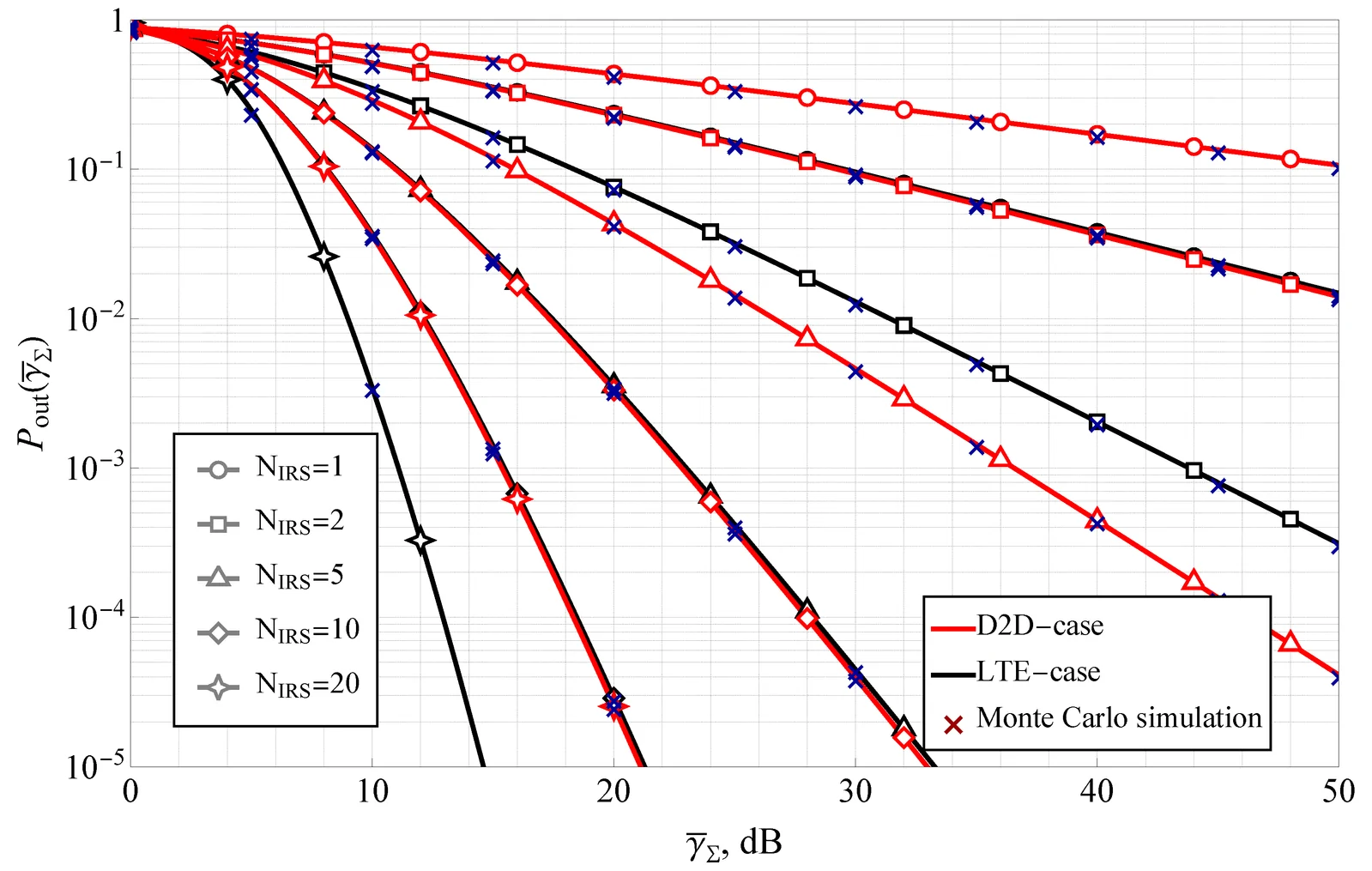
System reliability as a function of the output average SNR with $\gamma_{th}=3$ dB for various numbers of $N_{IRS}$.

**Figure 2:**
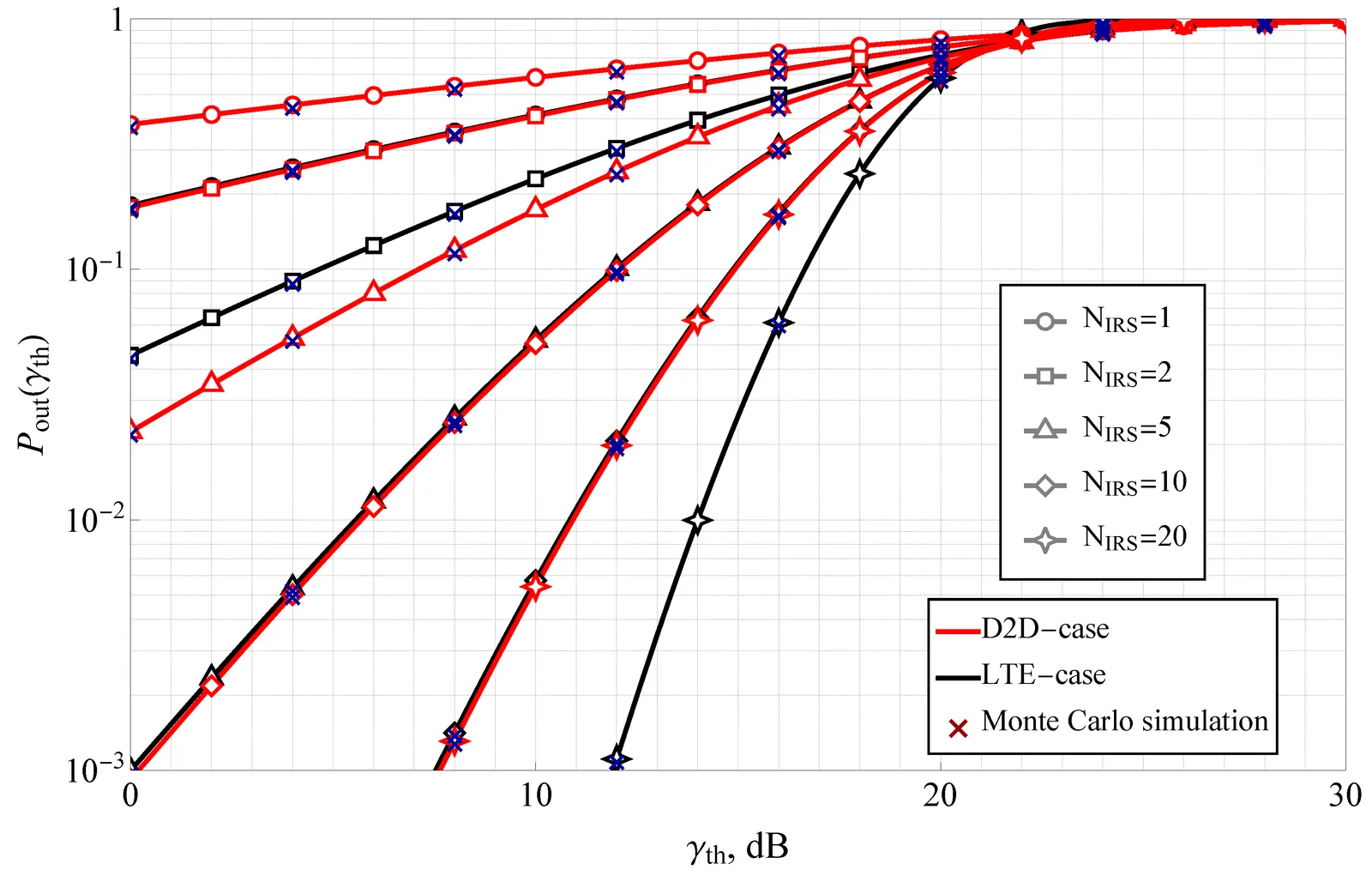
System reliability as a function of the outage threshold SNR with ${\bar{\gamma }}_{\Sigma }=20$ dB for various numbers of $N_{IRS}$.

**Figure 3:**
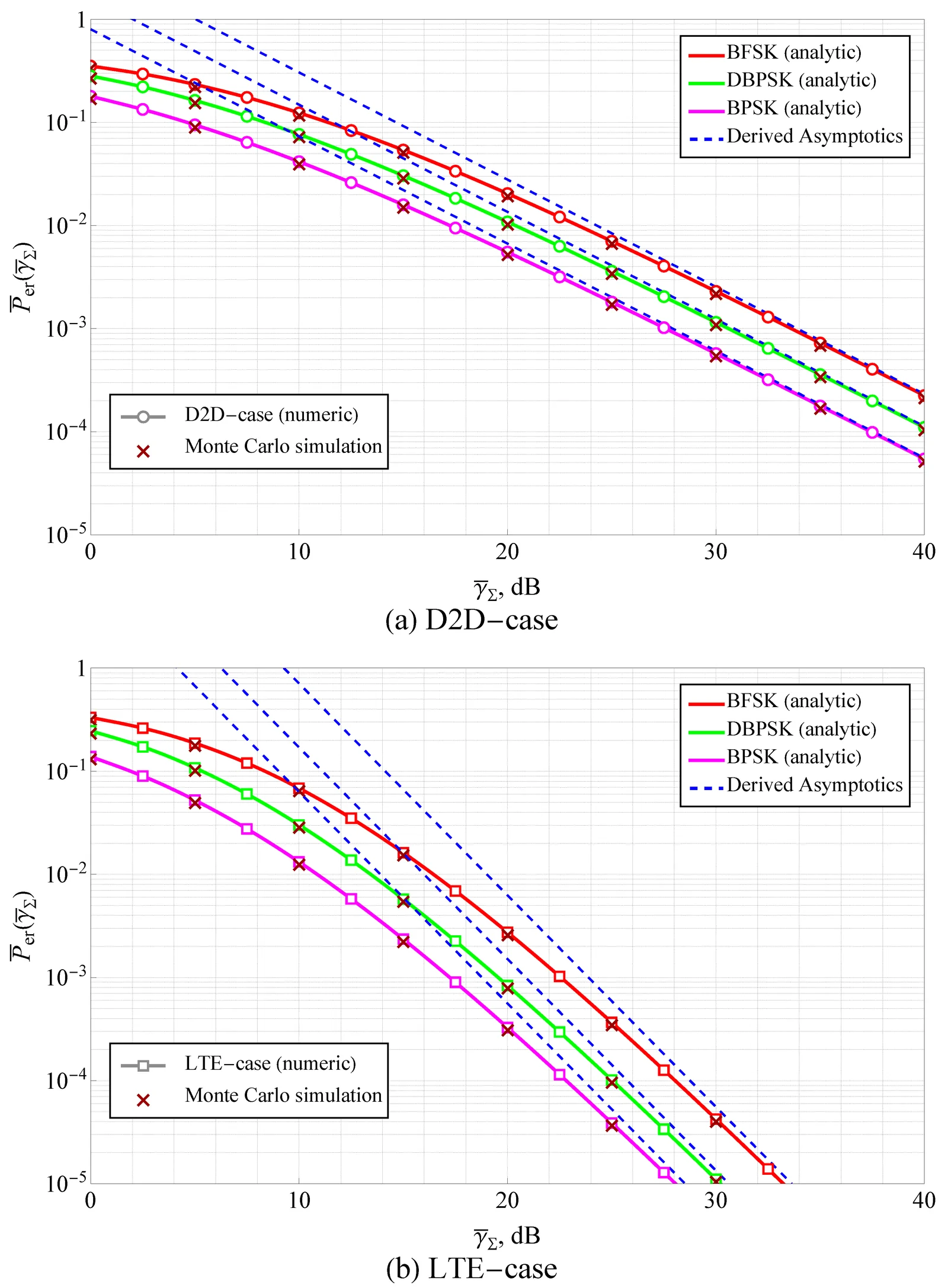
Average error rate comparison for coherent and non-coherent binary modulations.

**Figure 4:**
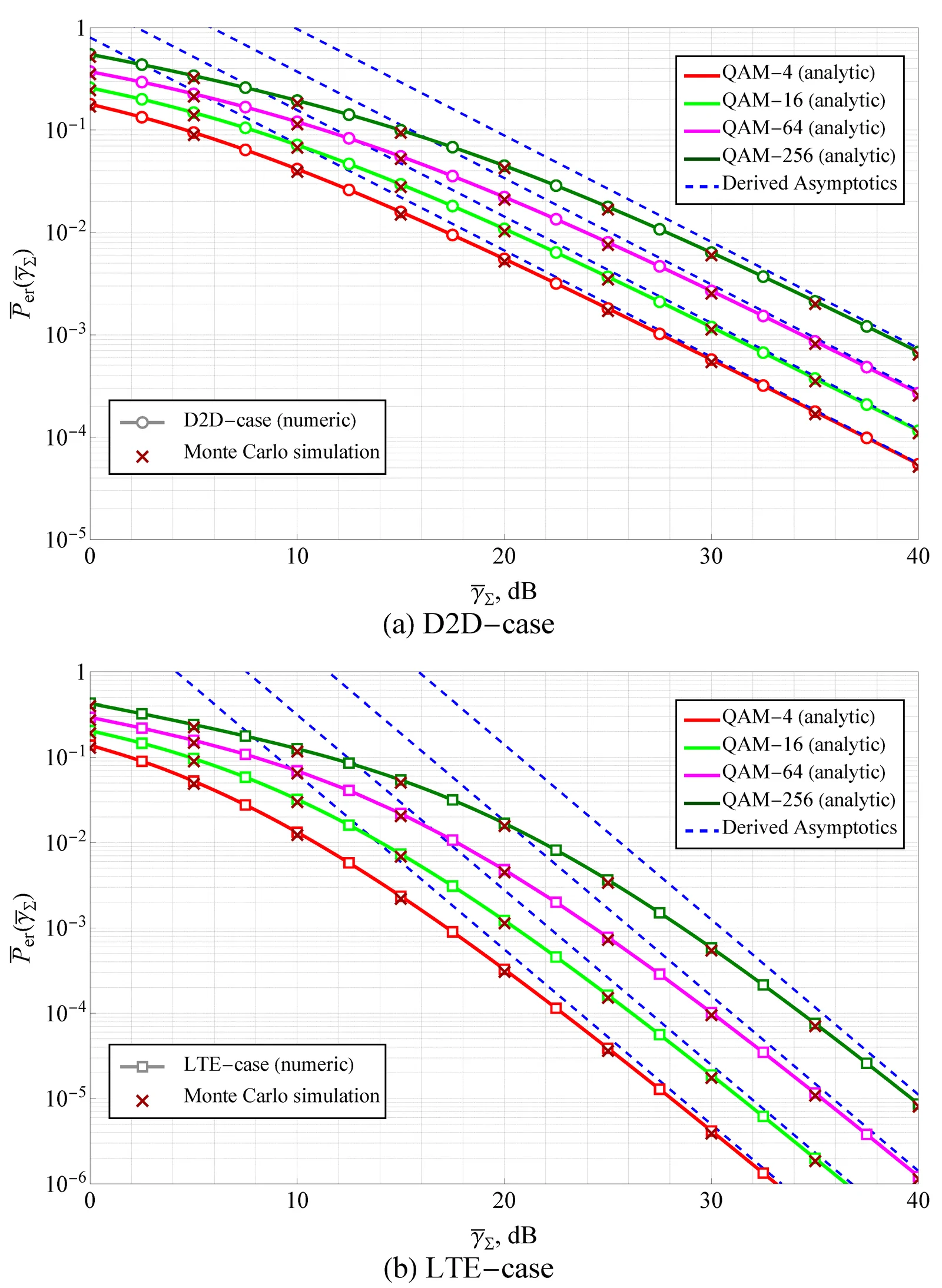
Impact of QAM constellation size on the average error rate for the case of $N_{IRS}=5$.

**Figure 5:**
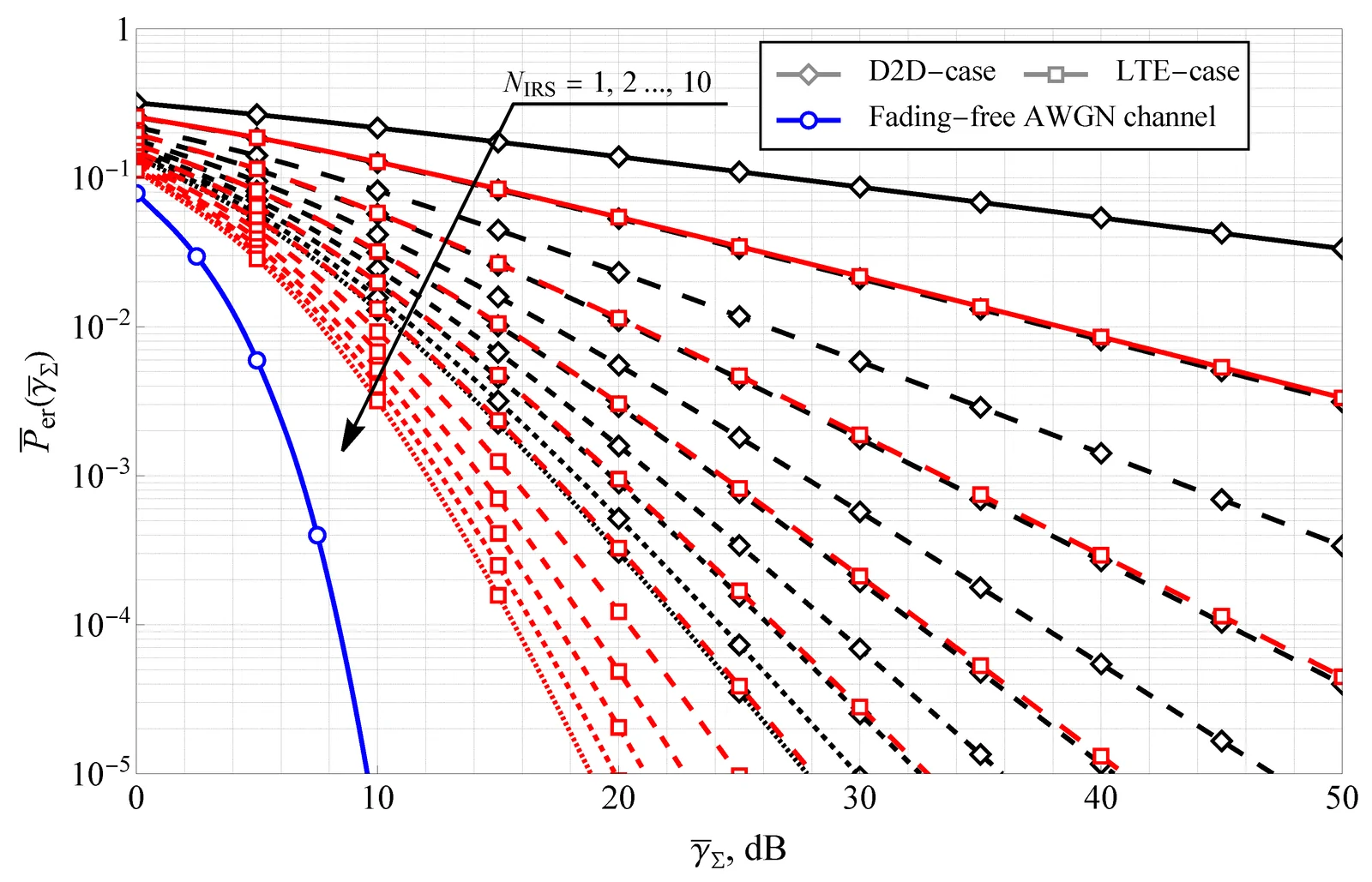
Impact of the number of IRS elements ($N_{IRS}$) on the average rate for the case of QPSK modulation. The red lines correspond to the LTE case, while the black lines correspond to D2D. The increase in dashing corresponds to an increase in the number of elements (from 1 to 10).

Furthermore, the section is divided into three consecutive parts: reliability analysis, quality analysis, and joint analysis. In Figure 1, Figure 2, Figure 3, Figure 4 and Figure 5, the results obtained via the derived expressions (presented in the plots with solid and dashed lines) are appended with their asymptotics (derived in the theoretical part and plotted with dashed straight blue lines), and the results obtained with the help of a Monte Carlo simulation (depicted by crossed markers).

### 5.1. Link Reliability Analysis

Figure 1 evaluates the reliability behavior of the IRS-assisted communication system through plotting the outage probability over a wide span of average received SNR. The effect of increasing IRS complexity is observable; the use of multiple reflective elements $N_{\mathrm{IRS}}$ improves the reliability performance significantly. As shown in the plot, the use of a single reflective element ($N_{\mathrm{IRS}}=1$) demonstrates an extremely slow decline in the outage probability due to the adverse effect of the extremely deep hyper-Rayleigh fading of the Inverse Power Lomax model. On the other hand, increasing the number of reflective elements to $N_{\mathrm{IRS}}=20$ increases the stability, enabling the LTE network to overcome the high reliability criterion of $10^{-5}$ at a relatively low average SNR of about 15 dB. A difference between the two cases persists, in which the LTE macrocellular scenario outperforms the short-range D2D scenario in terms of the lower value of outage probability at all average SNRs.

The results shown in Figure 2 demonstrate how the system reliability varies with respect to changes in the operational outage threshold SNR value, but for a fixed average received signal budget of 20 dB. Indeed, as expected from a purely qualitative standpoint, the outage probability exhibits a monotonic increase as the required threshold SNR value increases, as satisfying a more rigorous signal quality criterion becomes progressively more difficult for the receiver. Clearly visible is the increased resilience to channel fading provided by larger sizes of the reflecting surfaces, effectively widening the range of operation under challenging conditions of high threshold values. Specifically, for an intermediate target value of 10 dB, even the most basic setup with only $N_{\mathrm{IRS}}=1$ or 2 approaches complete outage, with outage probabilities above $10^{-1}$. On the other hand, the use of a modified reflecting surface with $N_{\mathrm{IRS}}=20$ achieves excellent reliability performance, keeping the outage probability around $10^{-3}$ for the LTE setup at a 10 dB threshold. It should be noted that the difference in the channel propagation models is evident during all threshold values considered, as illustrated by the systematic shift to the right of the LTE setup curves relative to the D2D scenario counterpart.

It is also interesting to note that some specific configurations deliver almost the same reliability for opposite propagation models, i.e., two-element IRS in the D2D-case performs as a one-element IRS in the LTE-case and so on. One can note that an IRS with some fixed number of elements in the LTE-case has the same outage probability as the doubled-in-size panel but in the D2D scenario.

These results yield some practical guidance for the design of threshold-aware link adaptation and quality-of-service management algorithms. For example, for system operators it is possible to make use of such curves by dynamically varying the decoding threshold, coding rate, or even changing the size of the constellation depending on the available number of active IRS units, in order to reduce any potential disruptions in service, which may move the link into the outage region. Moreover, the plot establishes a clear quantitative trade-off between energy consumption and hardware scaling, allowing network designers to effectively substitute active transmit power with passive spatial aperture to achieve targeted ultra-reliable low-latency communication (URLLC) benchmarks.

### 5.2. Link Quality Analysis

**Figure 6:**
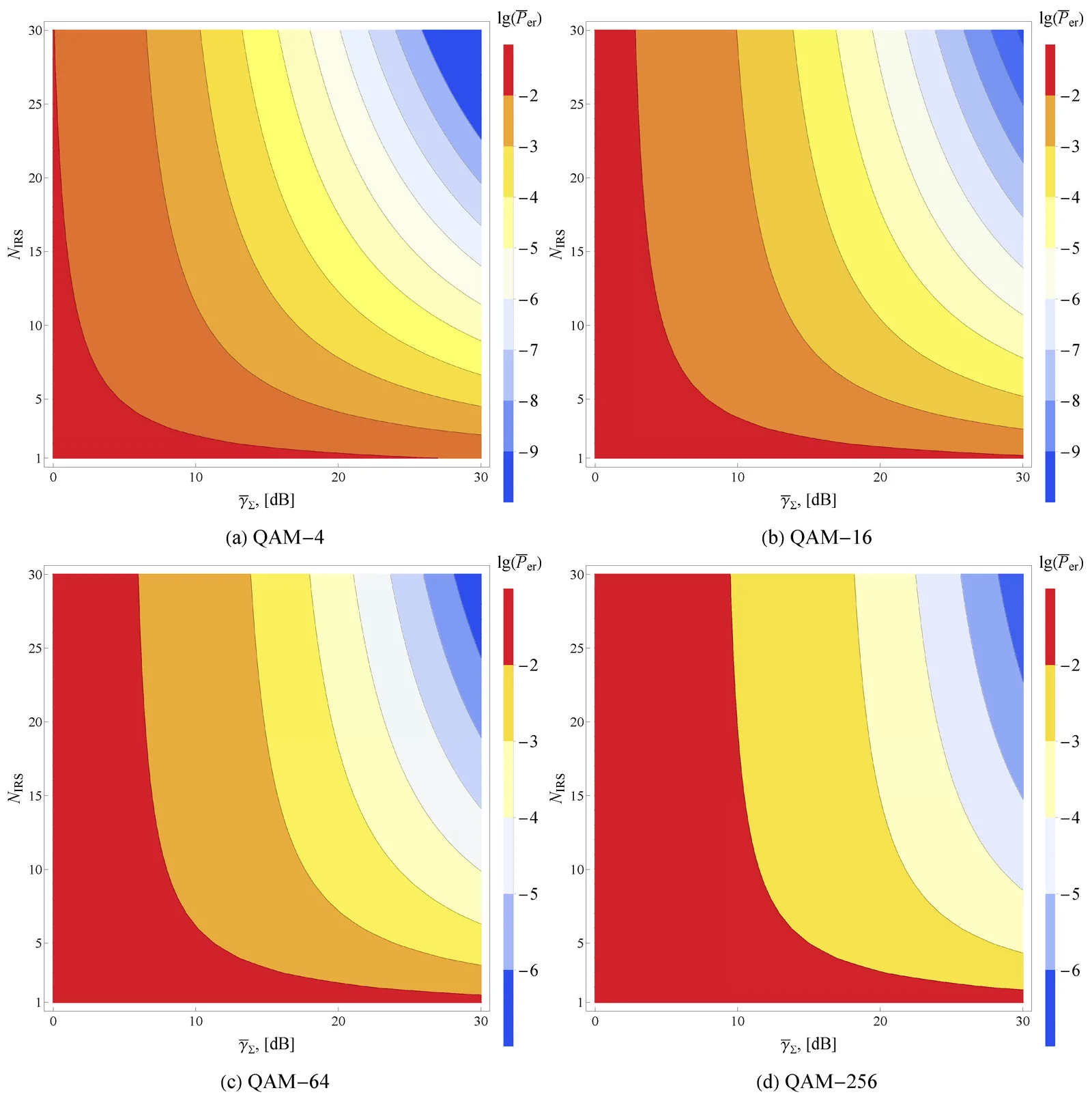
Contour plots of the joint impact of the number of IRS elements ($N_{IRS}$) and the average signal-to-noise ratio (${\bar{\gamma }}_{\Sigma }$) on the average error rate for QAM constellations with M={4,16,64,256} in the D2D-scenario.

Figure 3, Figure 4, Figure 5 and Figure 6 present the results of the average error rate analysis from the standpoint of 2D and 3D contour maps, which help to study the combined impact of several parameters simultaneously. Figure 3 and Figure 6 are plotted for a fixed IRS configuration with $N_{IRS}=5$ to study the impact of modulation type, constellation size, and IPL fading scenarios, whereas further plots depict the results for varying numbers of IRS elements, demonstrating their impact on the communication quality.

Figure 3 presents the average bit error rate as a function of the average received signal-to-noise ratio at the receiver (${\bar{\gamma }}_{\Sigma }$) in the range of [0,50] dB for three representative binary modulation formats, namely, binary frequency-shift keying (BFSK), differential binary phase-shift keying (DBPSK), and coherent binary phase-shift keying (BPSK), under two experimentally calibrated Inverse Power Lomax (IPL) fading scenarios assumed in the research.

Comparing the considered modulation formats, coherent BPSK consistently demonstrates superior performance, maintaining BER values approximately one order of magnitude lower than BFSK and half of that value for DBPSK across the entire assumed SNR range, which aligns with the well-established energy efficiency advantage of coherent detection architectures. The DBPSK curves occupy an intermediate position, reflecting the inherent penalty associated with differential encoding and non-coherent demodulation, while BFSK exhibits the most conservative reliability performance, requiring approximately 5–4 dB higher SNR to achieve equivalent BER targets relative to BPSK. A critical observation concerns the systematic separation between the LTE- and D2D-cases: for identical modulation and SNR values, the LTE-case curves remain consistently sufficiently lower, indicating a less severe effective fading severity attributable to the underlying IPL shape and scale parameters governing the composite cascaded channel statistics. The Monte Carlo simulation markers, represented by cross symbols, exhibit near-perfect coincidence with the derived analytical expressions across all modulation formats and fading scenarios, thus providing rigorous empirical validation of the proposed statistical approximation for the equivalent IRS-assisted channel model. Moreover, the high-SNR asymptotic expressions, depicted as dashed lines, demonstrate accurate convergence to the exact analytical curves once the system enters the moderate-to-high SNR regime (${\bar{\gamma }}_{\Sigma }\geq 15$ dB), whereas noticeable deviations persist in the low-SNR region due to the truncation of higher-order terms in the dominant-term asymptotic expansions.

The modulation-dependent performance penalty becomes particularly pronounced in the intermediate SNR region ($7.5\leq {\bar{\gamma }}_{\Sigma }\leq 15$ dB), where incremental SNR improvements of merely 2–3 dB yield BER reductions up to the order of magnitude, suggesting that adaptive modulation selection could substantially reduce transmit power requirements prior to necessitating IRS aperture expansion.

From a practical system design perspective, Figure 3 establishes quantitative benchmarks for modulation type selection (that is, coherent or non-coherent) under experimentally verified IPL fading. For example, it indicates that coherent BPSK should be prioritized in cases of energy-constrained applications with fixed BER (e.g., $\leq 10^{-5}$), whereas BFSK may suffice for deployments operating with moderate link quality thresholds. The figure also underscores the critical role of accurate channel parameter estimation, as mischaracterization of the IPL parameters $\left(\alpha_{i},\beta_{i}\right)$ could lead to substantial overestimation of achievable link quality, particularly in the hyper-Rayleigh regime where fading tails dominate system performance.

Figure 4 extends the presented analysis with the comparison of various coherent modulations, including square *M*-ary quadrature amplitude modulation (QAM) formats, specifically 4-QAM, 16-QAM, 64-QAM, and 256-QAM. It illustrates the fundamental trade-off between spectral efficiency and link reliability under IPL fading conditions. The BER curves exhibit strict monotonic ordering with respect to constellation size, with 4-QAM achieving the lowest error probability and 256-QAM demonstrating the highest sensitivity to fading and additive white Gaussian noise, a behavior that becomes increasingly pronounced as ${\bar{\gamma }}_{\Sigma }$ decreases below 25 dB (for high-order constellations). Quantitatively, transitioning from 4-QAM to 16-QAM incurs an approximate SNR penalty of 3–4 dB at BER = $10^{-4}$, whereas the progression from 64-QAM to 256-QAM demands an additional 5–7 dB, highlighting greater reliability degradation associated with dense constellation packing under hyper-Rayleigh fading. The LTE-case parameterization consistently outperforms the D2D-case across all QAM orders and SNR values. The increased performance gap for higher-order modulations suggests that severe fading environments disproportionately penalize spectrally efficient transmission schemes.

The agreement between analytical results, Monte Carlo simulations, and asymptotic approximations remains highly accurate throughout the investigated parameter space, validating not only the statistical approximation employed for the equivalent IRS-assisted channel but also the derived Meijer-*G* function-based expressions for average error probability. The asymptotic curves converge with increasing precision in the upper-SNR region, i.e., ${\bar{\gamma }}_{\Sigma }\geq 30$ dB.

The performed cross-comparison with Figure 3 demonstrated that the sensitivity to modulation order is substantially stronger than the choice of the reception scheme for binary modulation schemes (e.g., coherent or non-coherent); specifically, increasing the constellation order from 16-QAM to 64-QAM introduces a considerably larger penalty than the transition from DBPSK to coherent BPSK, indicating that high-order QAM requires substantially better channel conditions and/or larger IRS apertures to remain operationally feasible.

Another important observation is that the improvement associated with the D2D-case propagation conditions is insufficient to completely offset the reliability degradation introduced by dense constellations such as 256-QAM, implying that modulation penalty and fading penalty accumulate rather than compensate each other in severe propagation environments. From a system-design perspective, Figure 4 strongly supports the necessity of adaptive modulation and coding (AMC) strategies in IRS-assisted wireless systems operating under IPL fading conditions, where the lower-order QAM constellations seem to be significantly more robust in the presence of heavy-tailed fading, whereas very high-order constellations (e.g., 128-QAM and higher) become practical only when sufficiently large IRS gains and high average SNR values can simultaneously be achieved.

The plots, presented in Figure 4, also provide quantitative guidance for link adaptation algorithms: for instance, achieving BER $\leq 10^{-5}$ with 64-QAM under LTE-case fading requires ${\bar{\gamma }}_{\Sigma }\geq 35$ dB, whereas the same target with 4-QAM is attainable at ${\bar{\gamma }}_{\Sigma }\approx 28$ dB, suggesting that dynamic constellation switching could yield substantial power savings in fading conditions. Moreover, the approximately parallel high-SNR asymptotes across different QAM orders indicate that the diversity order remains invariant to constellation size, with the primary impact of higher-order modulation manifesting as a coding gain penalty rather than a reduction in diversity, a finding consistent with classical fading channel theory but here rigorously established for the IRS-assisted communications with an IPL fading channel model. Furthermore, the plot demonstrates that the threshold SNR (i.e., the SNR required for entering the asymptotic regime) increases with modulation order, implying that high-order QAM systems require more extensive averaging or larger IRS apertures to achieve predictable high-SNR behavior. From a practical implementation standpoint, Figure 4 indicates the importance of accurate channel state information (CSI) acquisition for high-order QAM (which is a common problem for modern IRS applications), as residual estimation errors could effectively shift the operating point toward the high-BER region, particularly under severe IPL fading where the channel exhibits heavy-tailed statistics.

After the careful and thorough analysis of the impact of modulation-related parameters (i.e., modulation type and constellation size) as well as the IPL channel fading scenarios, it is important to account for the achievable gain for various IRS panel sizes (i.e., the number of IRS elements).

Figure 5 illustrates the influence of $N_{\mathrm{IRS}}$ on the assumed link quality metric directly visualizing some type of “array-gain” mechanism predicted analytically in the theoretical section. The presented results give a better understanding of the possible scalability of IRS-assisted fading mitigation. The fading-free AWGN benchmark curve, depicted as a solid blue line, decreases extremely rapidly with SNR and represents the ideal performance limit achievable in the absence of fading (and without the IRS, i.e., with the direct link between the transmitter and the receiver), serving as a fundamental benchmark for quantifying the residual fading penalty under practical IRS configurations. The LTE-case and D2D-case curves corresponding to $N_{\mathrm{IRS}}=1,2,\ldots ,10$ gradually approach this benchmark as the IRS size increases, demonstrating that larger IRS apertures systematically improve the BER performance, which can be clearly explained by the enhanced spatial diversity. As demonstrated in Figure 3 and Figure 4, for a fixed number of IRS elements, the LTE-case constantly outperforms the D2D-case, although it is interesting to note that the ABER curves for some fixed $N_{IRS}$ in the LTE-case almost coincide with the curves in the D2D-case but with the doubled number of elements (e.g., ${\bar{P}}_{er}{|}_{LTE}^{N_{IRS}=5}={\bar{P}}_{er}{|}_{D2D}^{N_{IRS}=10}$). For small IRS sizes ($N_{\mathrm{IRS}}\leq 4$), the system performance remains relatively far from the AWGN benchmark, implying that a limited number of reflecting elements cannot completely compensate for the hyper-Rayleigh behavior of the IPL channel, whereas increasing $N_{\mathrm{IRS}}$ beyond 8 yields progressively diminishing returns, with the gap between the practical IRS-assisted system and the fading-free benchmark becoming asymptotically smaller. These numerical results support Corollary 1 derived in the theoretical section, which establishes that an infinitely large IRS completely eliminates fading effects, while finite IRS apertures introduce an effective SNR penalty factor of $\alpha_{g}/\left(\alpha_{g}+1\right)$. Figure 5 also demonstrates the overall shrinkage of the total effect from the increase of $N_{\mathrm{IRS}}$: increasing the number of elements from 2 to 4 (in the LTE-case) produces a substantially larger gain (approximately 18 dB SNR gain at BER $=10^{-4}$) than increasing it from 4 to 6 (approximately 7 dB gain). This suggests the existence of an optimal IRS size beyond which additional hardware complexity yields negligible performance benefits.

Moreover, the non-linear relationship between $N_{\mathrm{IRS}}$ and ABER improvement implies that IRS-assisted system design must account for such practical factors as hardware constraints, control overhead, and channel estimation complexity, as they can limit the achievable aperture size in real-world implementations.

A cross-analysis of Figure 3, Figure 4 and Figure 5 demonstrates the fact that the enhanced asymptotic accuracy, which is observed in a high-SNR region, can be explained by the evolution of the equivalent channel toward an effectively non-fading regime, with the IRS acting not simply as a passive signal-combining structure but, rather, as a statistical equalization mechanism capable of suppressing severe fading fluctuations. From an engineering standpoint, Figure 5 also underlines the importance of joint optimization of IRS size and transmit power, because (as follows from the joint analysis of Figure 3, Figure 4 and Figure 5) excessive reliance on IRS aperture expansion without proper SNR scaling may yield suboptimal energy efficiency; this can be especially critical in the scenarios with battery-constrained deployments (e.g., IoT, Industrial IoT, sensor networks, etc.).

**Figure 7:**
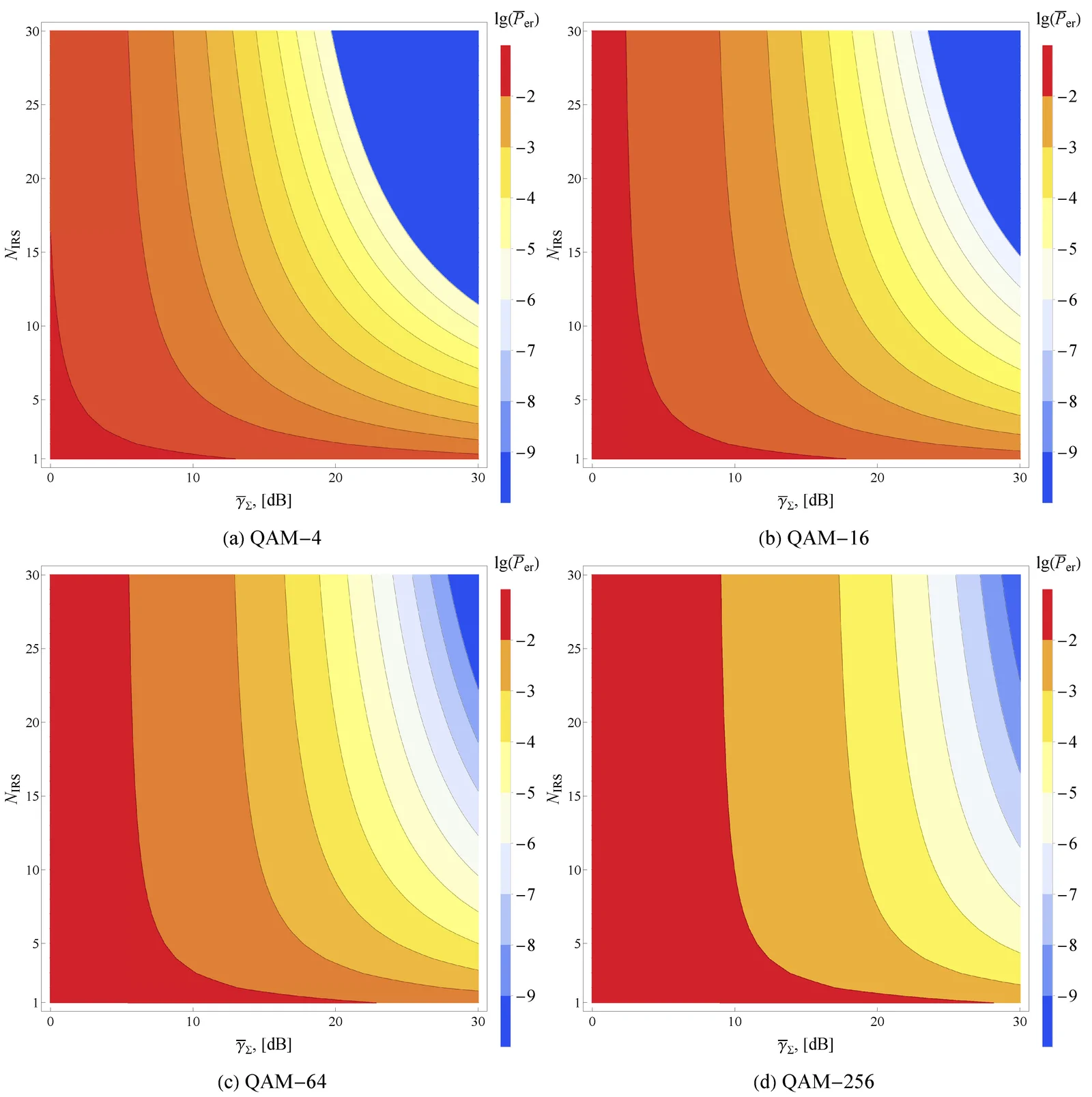
Contour plots of the joint impact of the number of IRS elements ($N_{IRS}$) and the average signal-to-noise ratio (${\bar{\gamma }}_{\Sigma }$) on the average error rate for QAM constellations with M={4,16,64,256} in the LTE-scenario.

As the impact of $N_{IRS}$ on the average error rate can be regarded as the penalty in the total SNR, their joint analysis is critical for a deeper understanding of the link quality. To assess such dependencies, Figure 6 and Figure 7 present the average error probability as a joint function of ${\bar{\gamma }}_{\Sigma }$ and $N_{\mathrm{IRS}}$ in terms of contour maps. The results are plotted for four QAM modulation orders (4-QAM, 16-QAM, 64-QAM, and 256-QAM) under the D2D-case (in Figure 6) or the LTE-case (in Figure 7) for IPL parameterization. Because for the assumed parameters’ values the range of error rate change is very large, the plotted values are logarithmically scaled, e.g., the contour line $\mathrm{lg}{\bar{P}}_{er}=-4$ corresponds to ${\bar{P}}_{er}=10^{-4}$. The lower-left corner of each subplot represents poor system performance characterized by low SNR and insufficient IRS gain, whereas the upper-right corner represents high-quality operation suitable for enhanced mobile broadband scenarios. The contour structure clearly demonstrates that increasing either the average SNR or the number of IRS elements improves performance, thus stating the multidimensional trade-off between the physical aperture size (in terms of $N_{IRS}$) in the spatial domain and the transmitted power allocation in the energy domain. The performed quantitative analysis of the contour-line trajectories shows that some prespecified target error rate (i.e., ${\bar{P}}_{\mathrm{er}}=10^{-3}$ or $\mathrm{lg}({\bar{P}}_{\mathrm{er}})=-3$ can be flexibly achieved through diverse combinations of the underlying system parameters. In the highly constrained spatial regime where $N_{\mathrm{IRS}}<5$, the contour lines exhibit a shallow horizontal gradient, signifying that additional power increase yields diminishing returns unless it is accompanied by an expansion of the reflective surface aperture to mitigate the severe fading. On the other hand, within the large-scale aperture regime where $N_{\mathrm{IRS}}>20$, the isolines undergo a sufficient change along with the increase of the average SNR, indicating that the composite channel asymptotically approaches a deterministic, fading-free operational state where performance becomes mainly power-driven.

Comparing Figure 7 with Figure 6, one can see that for the LTE-case the feasible operating region expands significantly, with the degradation becoming increasingly severe as the modulation order grows: while 4-QAM still achieves acceptable performance (e.g., $\log_{10}\left(P_{e}\right)\leq -3$) over a relatively broad operating region, the 256-QAM configuration remains highly sensitive to both fading severity and insufficient IRS aperture size, requiring ${\bar{\gamma }}_{\Sigma }\geq 25$ dB and $N_{\mathrm{IRS}}\geq 10$ to attain comparable link quality. The contour structures exhibit stronger curvature than in Figure 6, indicating that the effectiveness of additional IRS elements depends critically on the current operating point; in particular, the gain associated with increasing $N_{\mathrm{IRS}}$ becomes more substantial only once the system already operates in a sufficiently high-SNR regime (${\bar{\gamma }}_{\Sigma }\geq 15$ dB), suggesting that the IRS acts most efficiently when supporting a link with an already moderate power budget rather than attempting to rescue an extremely weak communication channel.

The cross analysis of the subplots for the assumed modulations (i.e., from 4-QAM to 256-QAM) for both cases (that is, D2D in Figure 6 and LTE in Figure 7) demonstrates the difference between the higher-frequency long-range macrocellular scenario and the lower-frequency short-range device-to-device layout. The contour lines associated with the short-range environment with more severe propagation conditions experience a tighter geometric compression toward the upper-right high-power, high-aperture corner, which is mathematically described by the smaller fading shape parameter $\alpha_{i}=0.45$ relative to the macrocellular value of $\alpha_{i}=0.59$. Furthermore, the highly regular, parallel alignment of the high-SNR asymptotes across the varying constellation sizes confirms that the spatial diversity order remains fundamentally invariant to the modulation density, acting purely as a horizontal coding gain shift, transforming a severely fading propagation environment into an additive white Gaussian noise equivalent link.

### 5.3. Joint Quality–Reliability Analysis

Finally, the results of the separate reliability and quality analysis can be collected via the assumed joint quality–reliability framework. To this end, the results of the analytical derivations presented in Theorems 4 and 5 were used to plot the parametric curves ${\bar{P}}_{er}\;=\;f\left(P_{out}\right)$ for different IRS sizes ($N_{\mathrm{IRS}}=1,2,4,8,16,32,64$) and two outage threshold values ($\gamma_{th}=0$ dB and 3 dB). Such an approach enables a comprehensive visualization of the trade-off between link quality and reliability of the IRS-assisted communications under the adopted IPL fading conditions. The bisection line serves as a reference separating regions dominated by reliability (above the bisector, where $P_{out}$ is suppressed more efficiently than ${\bar{P}}_{er}$) from those dominated by quality (below the bisector, where ${\bar{P}}_{er}$ decreases more rapidly than $P_{out}$). This approach helps to identify operating conditions that favor one metric over the other. First of all, one can clearly see the influence of the IRS size: increasing $N_{\mathrm{IRS}}$ systematically shifts the curves toward the upper-left region of the plot, corresponding to greater improvements of reliability rather than quality, which confirms the theoretical large-IRS asymptotic analysis presented in Theorem 3 and Corollary 1.

As was earlier noted, the comparison between the LTE-case and the D2D-case in separate quality or reliability analysis demonstrates the superior performance of the LTE-calibrated propagation environment. However, the joint analysis reveals some indirect implications, which cannot be captured by standalone study. For example, it is clear that for the same outage probability and IRS size, the D2D-case achieves smaller BER values than the LTE-case by approximately 0.5–1.5 orders of magnitude, highlighting the persistent influence of the underlying IPL parameters on the joint quality–reliability trade-off. This is mainly due to the fact that for the JQR curve, the outage probability and average error rate are evaluated for the same average signal-to-noise ratio, which leads to such behavior.

**Figure 8:**
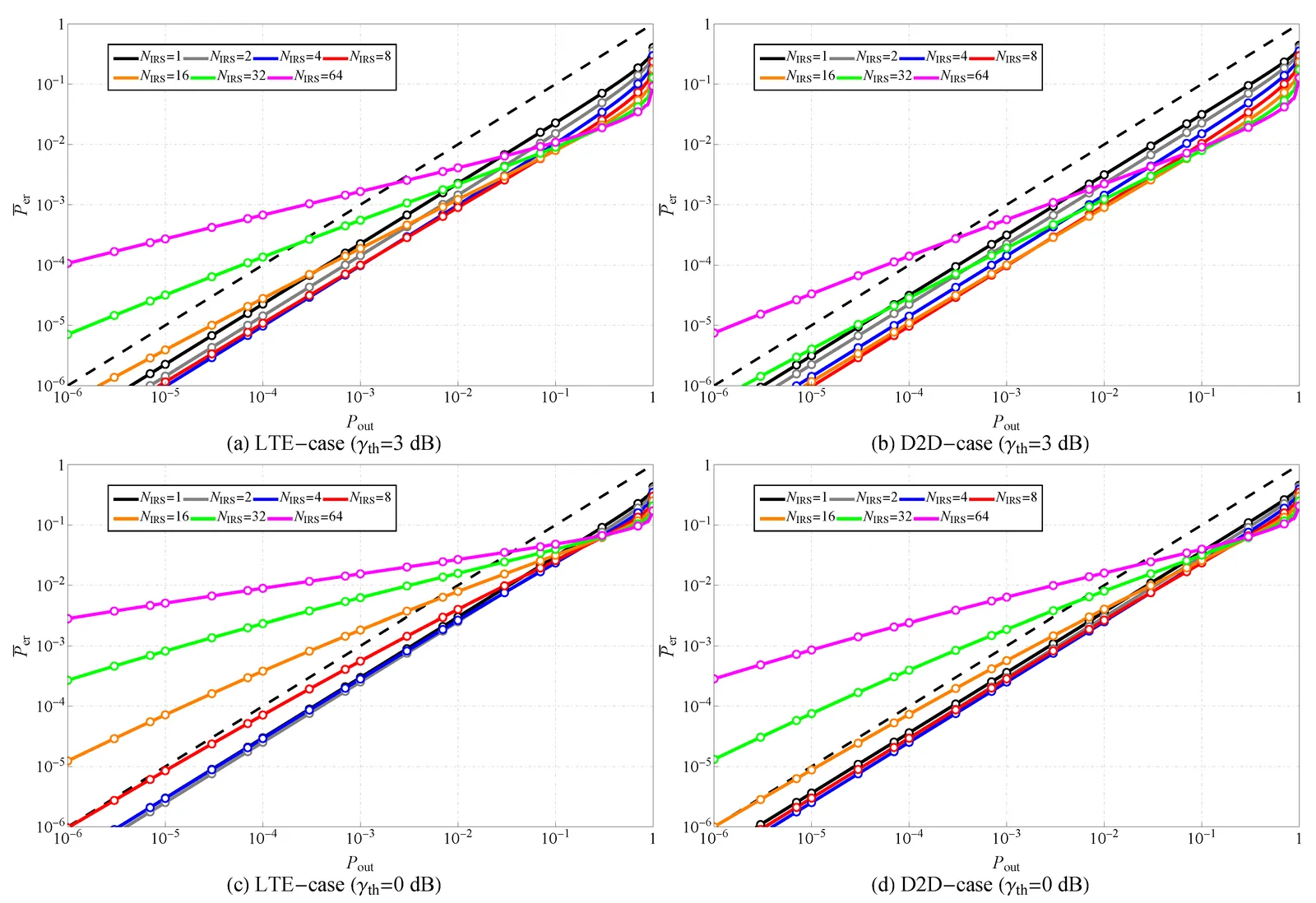
Joint quality–reliability analysis for QAM-4 modulation.

An interesting observation regards the role of the outage threshold $\gamma_{th}$. Lowering this value from 3 dB to 0 dB results in moving the corresponding JQR curve to a more favorable working area, showing that the sensitivity to channel degradation is reduced when using a less stringent outage constraint. From the practical viewpoint, the presented results show the potential for adaptive thresholds in the development of QoS-aware algorithms for allocating resources to wireless communications. In addition, the nonlinear nature of the JQR curves for the low-outage regimes is evident from the obtained curves (see Figure 8). Indeed, when deploying sufficiently large intelligent reflecting surfaces ($N_{\mathrm{IRS}}\;\geq \;16$), even slight improvements in the outage probability translate into significant BER enhancements, while for small IRS apertures ($N_{\mathrm{IRS}}\leq 4$), the JQR curves are located close to the bisector, thus confirming that limited IRS apertures cannot decouple quality from reliability. Cross-examination of Figure 8 with Figure 1, Figure 2, Figure 3, Figure 4, Figure 5, Figure 6 and Figure 7 proves the coexistence of the phenomena considered earlier such as modulation-related penalties, fading severity depending on the channel realization, and fading suppression due to the presence of IRS in the communication link. Modulation-related penalties, fading severity dependent on the channel realization, and fading suppression induced by IRS determine the final position on the quality–reliability plane. Importantly, it follows from the presented results that IRS-assisted wireless systems allow adjustment of the trade-off between quality and reliability, thus offering a promising solution for supporting URLLC and mMTC services in the IPL environment. With regard to practical implementation, the use of the JQR diagram allows establishment of the region in which an IRS-assisted communication link either focuses on reliability (with minor quality losses) or vice versa, as proposed theoretically in Section 3. For example, the region above the bisector for $N_{\mathrm{IRS}}=64$ and $\gamma_{th}=0$ dB shows that the improvements in reliability can be achieved without compromising the quality.

### 5.4. JQR Sensitivity Analysis

#### 5.4.1. JQR Parity Sensitivity to Threshold SNR

**Figure 9:**
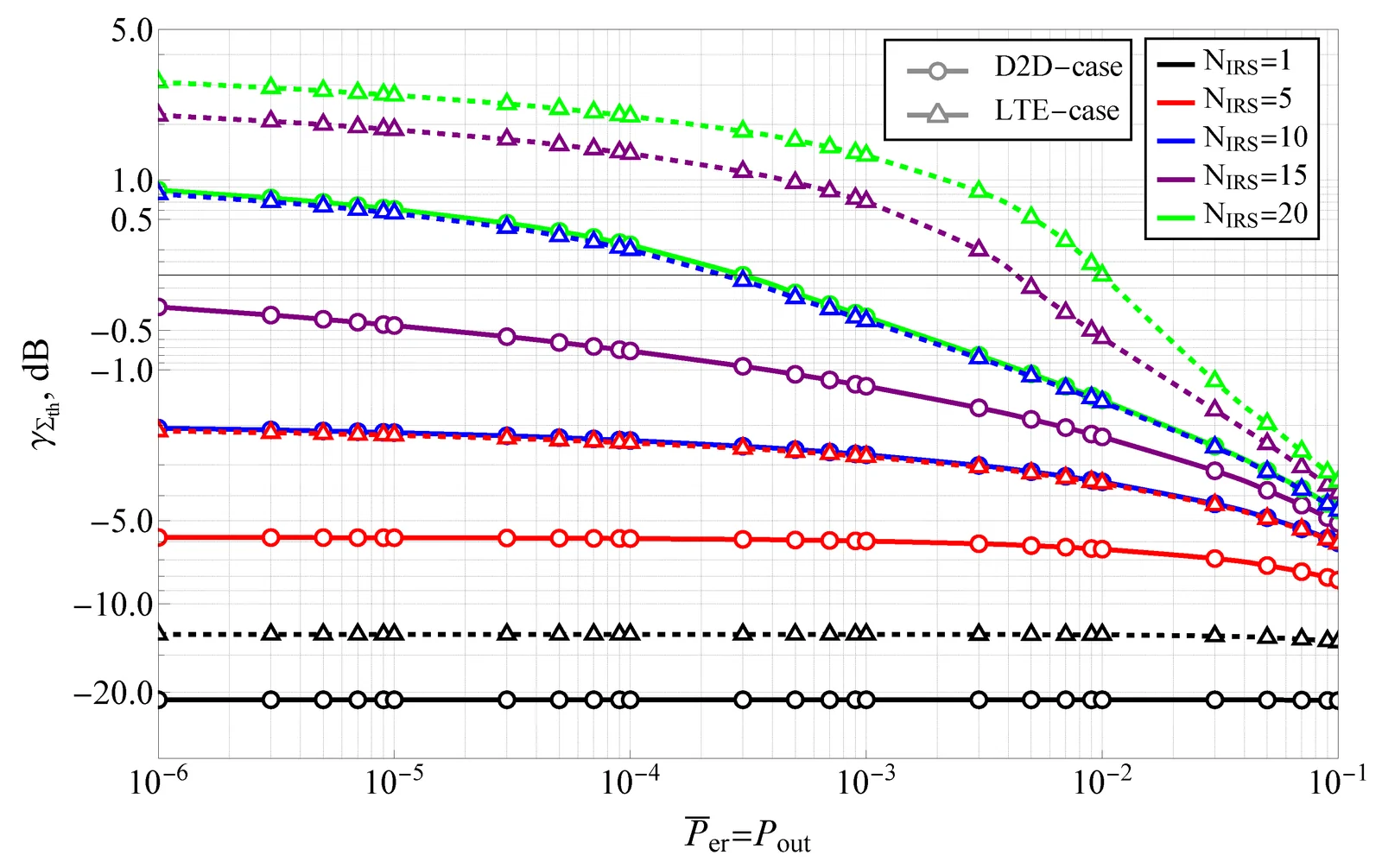
Total output SNR threshold required to achieve the prescribed parity level as a function of $N_{IRS}$.

Because, as was noted, the JQR parity line depends on the threshold SNR $\gamma_{\Sigma_{th}}$, its sensitivity issues must be addressed. Figure 9 complements the parametric JQR curves of Figure 8 by directly visualizing the required total output SNR threshold $\gamma_{\Sigma_{th}}$ needed to achieve a prescribed parity level ${\bar{P}}_{er}=P_{out}$ (i.e., the exact intersection point with the bisector of Figure 8), plotted as a function of the target parity level for $N_{\mathrm{IRS}}\in \left\{1,5,10,15,20\right\}$ for both specific propagation scenarios (i.e., the LTE-case and the D2D-case). It can be noted that $\gamma_{\Sigma_{th}}$ decreases monotonically with $N_{\mathrm{IRS}}$, confirming that larger IRS apertures reduce the SNR budget required to reach any given joint quality–reliability parity target. For instance, achieving parity at the $10^{-3}$ level requires approximately 5–6 dB less SNR when increasing $N_{\mathrm{IRS}}$ from 5 to 10. In the given range of values, the LTE-case curves lie always below their D2D-case counterparts for the same $N_{\mathrm{IRS}}$. This helps to conclude the persistent advantage of the LTE-calibrated propagation environment observed in Section 5. It is important to highlight that the curves flatten substantially for very small parity levels ($P_{er}=P_{out}≲10^{-4}$), which essentially means that the threshold SNR becomes insensitive to the required parity level. This indicates that once the system is driven deep into the high-reliability/high-quality region, further tightening of the joint target imposes a comparatively modest additional SNR penalty. Such an observation is practically useful for setting realistic URLLC-oriented parity targets without incurring excessive power overhead.

#### 5.4.2. SNR Penalty Factor Sensitivity to Possible IRS Phase Errors

Another critical factor that has to be taken into account in practice is the possible non-ideal phase compensation at the IRS [55,56,57,58]. Even though, as was explicitly stated in the preliminaries, this specific case is out of the direct scope of the research, some analysis must be performed. To address the practical concern of the influence of imperfect IRS phase control on the obtained results, a numerical simulation was carried out. First, it should be noted that all the assumed performance metrics are given in terms of the approximating gamma distribution shape parameter $\alpha_{g}$, which itself is evaluated via the first- and second-order moments of the output envelope at the receiver. Thus, we assume the same model as in Section 2.1 but with the following modification: $\arg\left\{{\dot{\rho }}_{l}\right\}=\arg\left\{{\dot{h}}_{1,l}\right\}+\arg\left\{{\dot{h}}_{2,l}\right\}+\varphi_{l}$, where $\varphi_{l}$ is the residual phase error due to non-ideal phase compensation. Utilizing the prevailing Von Mises model [55], the probability density function for any *l* will be given by [59]

(44) $$p_{\Phi }\left(\varphi \right)=\frac{e^{\kappa \cos\varphi }}{2\pi I_{0}\left(\kappa \right)},\;\varphi \in \left[-\pi ,\pi \right),$$

where κ≥0 is the concentration parameter and $I_{0}\left(\cdot \right)$ is the modified Bessel function of the first kind of order zero. This distribution is the natural circular analogue of the Gaussian and reduces to a perfectly phase-aligned IRS as κ→∞ and to a fully randomized (uniformly distributed) reflection phase as κ→0.

The circular variance associated with this phase-error model [59] can be obtained with the help of the characteristic function $E[e^{j\varphi }]$ of the Von Mises distribution:

(45) $$\sigma_{\varphi }^{2}≜1-E\left[\cos\varphi \right]=1-\frac{I_{1}\left(\kappa \right)}{I_{0}\left(\kappa \right)},$$

which serves as the natural sensitivity parameter, as the aggregate coherent combining gain of the cascaded channel is attenuated by some factor. Repeating the moment-matching procedure of Section 3 under this phase-error model yields the degraded shape parameter

(46) $$\alpha_{g}\left(\kappa \right)\approx {\left(1-\sigma_{\varphi }^{2}\left(\kappa \right)\right)}^{2}\;\alpha_{g},$$

where $\alpha_{g}$ denotes the ideal (phase-error-free) moment-matched shape parameter derived in Section 3.

**Figure 10:**
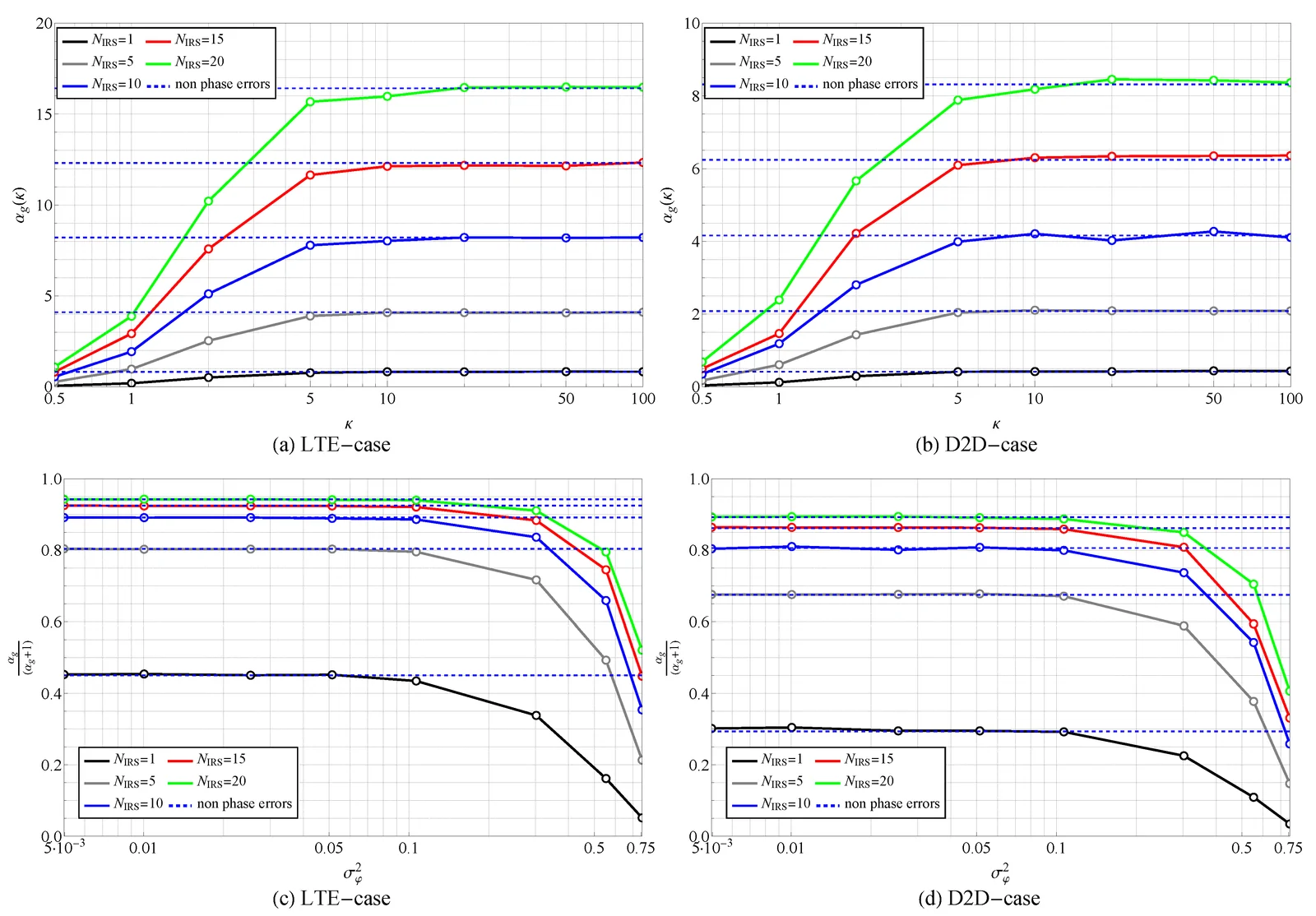
The impact of the residual IRS phase noise on the system parameters: (**a**,**b**) The impact of von Mises concentration parameter κ on the scale parameter $\alpha_{g}$. (**c**,**d**) The impact of residual IRS phase noise variance on SNR penalty factor $\frac{\alpha_{g}}{\alpha_{g}+1}$.

Figure 10a,b present the empirically estimated $\alpha_{g}\left(\kappa \right)$ as a function of the Von Mises concentration parameter κ for $N_{\mathrm{IRS}}\in \left\{1,5,10,15,20\right\}$ under the LTE- and D2D-case parameterizations, respectively. The dashed horizontal lines denote the ideal (phase-error-free) asymptotic values of $\alpha_{g}$, which are correctly approached as κ→∞ for every $N_{\mathrm{IRS}}$, which demonstrates the asymptotic consistency with the derived analytical expression. As κ decreases below approximately 5 (equivalently, $\sigma_{\varphi }^{2}≳0.1$), $\alpha_{g}\left(\kappa \right)$ departs increasingly from its ideal saturation level, with the departure becoming progressively more pronounced for larger $N_{\mathrm{IRS}}$: because $\alpha_{g}$ scales linearly with $N_{\mathrm{IRS}}$ in the phase-error-free case, a fixed multiplicative penalty ${\left(1-\sigma_{\varphi }^{2}\right)}^{2}$ translates into a proportionally larger absolute loss of shape parameter for larger arrays. These results demonstrate that the fading-elimination benefit predicted for $N_{\mathrm{IRS}}\to \infty$ in Theorem 3 is increasingly fragile in the presence of realistic hardware phase noise.

As was demonstrated analytically, the average error rates (for both coherent and non-coherent modulations) asymptotically (for high SNR) exhibit a specific SNR penalty factor, which is given in terms of the ratio $\alpha_{g}/\left(\alpha_{g}+1\right)$. Thus, Figure 10c,d translates this effect directly into this factor plotted against the circular phase-error variance $\sigma_{\varphi }^{2}$. The penalty factor remains close to its ideal ceiling for well-controlled phase errors ($\sigma_{\varphi }^{2}≲0.05$, typical of moderate-resolution phase quantization, if the residual error resides from the quantization procedure), but degrades sharply once $\sigma_{\varphi }^{2}≳0.3$, particularly for larger $N_{\mathrm{IRS}}$, where the relative drop in the penalty factor is markedly steeper than for small arrays. This demonstrates that, while the asymptotic fading-elimination limit $\alpha_{g}/\left(\alpha_{g}+1\right)\to 1$ as $N_{\mathrm{IRS}}\to \infty$ is still technically attained for any fixed $\sigma_{\varphi }^{2}>0$, the practically achievable SNR gain for any finite deployment is strictly bounded away from this ideal limit by the multiplicative factor ${\left(1-\sigma_{\varphi }^{2}\right)}^{2}$. These insights indicate that residual hardware phase noise imposes practical, non-idealized ceilings on the achievable benefit of scaling up $N_{\mathrm{IRS}}$, and should be taken into account when translating the asymptotic theoretical results of Theorem 3 into realistic system design guidelines.

## 6. Discussion: Results Restrictions and Further Research Directions

The present analysis is confined to a static, terrestrial, two-dimensional IRS-assisted link, in which the transmitter, the reflecting surface, and the receiver are fixed (or move slowly compared to link operational time) and the channel statistics are described by a time-invariant IPL fading model. The evolution toward 6G networks, however, is expected to rely increasingly on three-dimensional, aerial–terrestrial integrated architectures, combining terrestrial base stations, unmanned aerial vehicles (UAVs), high-altitude platforms (HAPS), and low-Earth-orbit satellites within a single coordinated communication layer. In such environments, node mobility in three spatial dimensions, elevation-angle-dependent line-of-sight probability, and rapidly time-varying multipath conditions render a single static fading model (e.g., the IPL distribution assumed herein) insufficient on its own to characterize the end-to-end channel. Thus, a dynamically adaptive, unified channel-model-driven framework becomes necessary to preserve link stability under such extreme 3D mobility conditions.

A representative example of this emerging direction is provided by the stacked intelligent metasurface (SIM)-empowered framework for low-altitude intelligent networks (LAINs) proposed in [60], in which a unified channel model is jointly used to drive the optimization of the reconfigurable surface configuration itself, rather than being treated as a fixed statistical input to an otherwise static performance analysis. This joint channel-model-driven optimization paradigm illustrates the type of methodology that would be required to extend the joint quality–reliability (JQR) framework developed in the present work to 3D aerial–terrestrial scenarios.

Extending the proposed JQR methodology in this direction would require, at minimum, the following: (i) Replacing the static IPL fading assumption with a spatially and temporally varying channel model capturing elevation-dependent line-of-sight probability and Doppler effects induced by 3D node mobility. (ii) Extending the proposed formulation (with single IRS panel) to the case of multiple IRS panels installed on different mobile nodes with multilayer/stacked configurations of beyond diagonal architecture. (iii) Including 3D geometry and system information into the reformulated expressions for the outage probability and average error rate metrics, so that the resulting JQR curves could be used within a real-time, channel-model-driven optimization loop rather than as an offline design tool.

Moreover, such complex configurations, including possible multiple IRSs, large velocities, fast-changing environment (thus fading), and large-scale multilayer IRS architectures, require highly intricate adaptation algorithms (especially when channel state information is estimated at the IRS). This eventually leads to highly demanding processing procedures and, thus, power-consuming hardware. Nevertheless, recent research [61,62] demonstrates that the application of machine learning and artificial intelligence algorithms can effectively level down the complexity of the problem by the joint utilization of ad hoc neural networks adapted for beamforming [63,64], channel estimation [65,66], signal detection [67], etc., which can be successfully achieved by, for example, federated learning. In this aspect, the JQR analysis presented in the current research can be of possible interest for further integration into the ML-based approach as it naturally combines several link performance metrics, thus delivering novel optimization strategies.

## 7. Conclusions

The research examines the joint quality–reliability characteristics of an IRS-assisted wireless communication system affected by multipath fading and shadowing based on Inverse Power Lomax distribution. The channel parameters derived from two different experimental propagation models are considered: the high-frequency, long-distance cellular communication model (LTE scenario) and the low-frequency, short-distance device-to-device scenario. Analytical closed-form solutions of outage probability (performance measure related to reliability) and average BER of coherent/non-coherent modulation schemes (performance measures related to quality) are obtained for both propagation scenarios. Based on the latter results, the joint performance curves and their asymptotic expressions at high signal-to-noise ratio (SNR) and at large IRS element counts are developed.

It is shown analytically and numerically that the infinite number of IRS elements allows complete elimination of fading effects, thus making the cascaded fading channel equivalent to an AWGN channel. For a finite number of IRS elements, the IRS adds a penalty factor affecting the average SNR. The formula for the latter is presented in the closed form. An important insight obtained from numerical analysis is that the LTE scenario exhibits superior performance compared to the D2D scenario due to the different IPL shape and scale parameters. Finally, the joint performance analysis shows two different operating regions in which the performance metrics dominate each other.

All the derived expressions and their interrelations have been extensively verified via simulations using the Monte Carlo technique, proving the exactness of the analytical derivations. To the best of the authors’ knowledge, the present paper fills a gap in the IRS communication systems research by studying a novel fading scenario (Inverse Power Lomax) and developing the approach that can be used as a practical tool for designing efficient communication systems under empirically verified conditions. Further research can include analysis of non-ideal IRS phase control and residual estimation errors, as well as multiuser IRS-assisted channels.

## Figures and Tables

**Table 1:** Simulation setup.

Parameter	Parameter Value
IRS size ($N_{IRS}$)	1…64
Modulation type	coherent/non-coherent
Modulation constellation size (*M*)	from BPSK to 256-QAM
Average output signal-to-noise ratio (${\bar{\gamma }}_{\Sigma }$, dB)	0…70
Propagation scenarios	D2D and LTE
Fading channel parameters for D2D-case	$\alpha_{D2D}=0.45$, $\beta_{D2D}=1.55$
Fading channel parameters for LTE-case	$\alpha_{LTE}=0.59$, $\beta_{LTE}=1.77$
Monte Carlo sample size	$5\cdot 10^{7}$

## Data Availability

No new data were created or analyzed in this study. Data sharing is not applicable to this article.
